# Ecological Overlap and Horizontal Gene Transfer in *Staphylococcus aureus* and *Staphylococcus epidermidis*

**DOI:** 10.1093/gbe/evv066

**Published:** 2015-04-16

**Authors:** Guillaume Méric, Maria Miragaia, Mark de Been, Koji Yahara, Ben Pascoe, Leonardos Mageiros, Jane Mikhail, Llinos G. Harris, Thomas S. Wilkinson, Joana Rolo, Sarah Lamble, James E. Bray, Keith A. Jolley, William P. Hanage, Rory Bowden, Martin C.J. Maiden, Dietrich Mack, Hermínia de Lencastre, Edward J. Feil, Jukka Corander, Samuel K. Sheppard

**Affiliations:** ^1^College of Medicine, Institute of Life Science, Swansea University, United Kingdom; ^2^Laboratory of Molecular Genetics, Instituto de Tecnologia Química e Biológica António Xavier (ITQB), Universidade Nova de Lisboa, Oeiras, Portugal; ^3^Laboratory of Bacterial Evolution and Molecular Epidemiology, Instituto de Tecnologia Química e Biológica António Xavier (ITQB), Universidade Nova de Lisboa, Oeiras, Portugal; ^4^Department of Medical Microbiology, University Medical Center Utrecht, The Netherlands; ^5^The Biostatistics Center, Kurume University, Fukuoka, Japan; ^6^MRC CLIMB Consortium, Institute of Life Science, Swansea University, United Kingdom; ^7^Wellcome Trust Centre for Human Genetics, University of Oxford, United Kingdom; ^8^Department of Zoology, University of Oxford, United Kingdom; ^9^Center for Communicable Disease Dynamics, Harvard School of Public Health; ^10^Bioscientia Labor Ingelheim, Institut für Medizinische Diagnostik GmbH, Ingelheim, Germany; ^11^Laboratory of Microbiology and Infectious Diseases, The Rockefeller University, New York; ^12^Department of Biology and Biochemistry, University of Bath, United Kingdom; ^13^Department of Mathematics and Statistics, University of Helsinki, Finland

**Keywords:** *Staphylococcus*, evolution, ecology, recombination, nosocomial infections

## Abstract

The opportunistic pathogens *Staphylococcus aureus* and *Staphylococcus epidermidis* represent major causes of severe nosocomial infection, and are associated with high levels of mortality and morbidity worldwide. These species are both common commensals on the human skin and in the nasal pharynx, but are genetically distinct, differing at 24% average nucleotide divergence in 1,478 core genes. To better understand the genome dynamics of these ecologically similar staphylococcal species, we carried out a comparative analysis of 324 *S. aureus* and S. *epidermidis* genomes, including 83 novel *S. epidermidis* sequences. A reference pan-genome approach and whole genome multilocus-sequence typing revealed that around half of the genome was shared between the species. Based on a BratNextGen analysis, homologous recombination was found to have impacted on 40% of the core genes in *S. epidermidis,* but on only 24% of the core genes in *S. aureus*. Homologous recombination between the species is rare, with a maximum of nine gene alleles shared between any two *S. epidermidis* and *S. aureus* isolates. In contrast, there was considerable interspecies admixture of mobile elements, in particular genes associated with the SaPIn1 pathogenicity island, metal detoxification, and the methicillin-resistance island SCC*mec*. Our data and analysis provide a context for considering the nature of recombinational boundaries between *S. aureus* and *S. epidermidis* and, the selective forces that influence realized recombination between these species.

## Introduction

Nosocomial infections are a worldwide public health problem. A recent survey estimated that 6.4% of UK patients suffer from hospital-acquired infections, rising to 23.4% in intensive care units (Hopkins et al. 2012). Among the most frequent causes are bacteria belonging to the genus *Staphylococcus*, in particular *Staphylococcus aureus* and *Staphylococcus epidermidis*. These organisms are common components of the commensal human microbiota, inhabiting skin and mucous membranes of healthy individuals, but also causing infection, typically connected with invasive surgery, catheterization, and wounds. Morbidity and mortality associated with these organisms is a major clinical challenge (Naber 2009), particularly because widespread resistance to β-lactam antibiotics reduces the range of treatments available, and the current annual cost to European health services has been estimated at 44 million euros (de Kraker et al. 2011).

The range of pathologies associated with nosocomial staphylococcal infection include pneumonia, septicemia, pyogenic infections (endocarditis, septic arthritis, and osteomyelitis), food poisoning, and surgical wound infections (Fraser and Spiteri 2011). *Staphylococcus epidermidis* is particularly associated with the increased use of indwelling medical devices such as artificial heart valves, prosthetic joints, and vascular catheters which provide a substrate for biofilm formation (del Pozo and Patel 2007; Mack et al. 2013). Differences in the infection biology of *S. aureus* and *S. epidermidis* may reflect different ecologies in the commensal environment. For example, the ubiquity of *S. epidermidi*s on the skin compared to *S. aureus,* which is more associated with the anterior nares (van Belkum et al. 2009; Frank et al. 2010; Sollid et al. 2014), may account for its proliferation in high dependency environments such as intensive care and burns units (Fraser and Spiteri 2011). However, there is also evidence that some strains of both species are more common than expected in the hospital environment, consistent with local expansions (Dominguez et al. 1994; Miragaia et al. 2007).

Molecular typing techniques, such as multilocus sequence typing (MLST) have been instructive in showing that *S. aureus* and *S. epidermidis* populations are different and highly structured with related lineages grouped into genotype clusters, or clonal complexes (Melles et al. 2007; Miragaia et al. 2007). This genetic variation reflects the action of mutation and horizontal gene transfer (Feil et al. 2003; Vos and Didelot 2009; Didelot and Maiden 2010). Although the clonal structure is consistent with the proliferation of successful lineages, the role of adaptation is difficult to quantify and it is not known to what extent intra- and interspecies genetic diversity reflects ecological differentiation.

For species and lineages to remain distinct requires that there are barriers to genetic exchange between them. Perhaps, the simplest type of barrier is in allopatry, where spatially isolated lineages expand and give rise to local variants. Such a model may apply to within-host evolution (Golubchik et al. 2013) as well as broader phylogeographic patterns (Ruimy et al. 2009). However, multiple lineages of both *S. aureus* and *S. epidermidis* can be isolated from the same patient (Hedin 1996; Ueta et al. 2007; Golubchik et al. 2013) and in this case ecological differences may be significant in the coexistence of species and lineages occupying discrete subniches (Otto 2009; Conlan et al. 2012).

In this study, we draw direct comparison between the genomes of *S. aureus* and *S. epidermidis* - mostly of clinical origin*,* to investigate the genetic differentiation between these distinct species with similar ecologies. Using a reference pan genome approach (Meric et al. 2014), we investigate core and accessory genome variation, and realized recombination within- and between-species. Genome-wide signatures of genetic differentiation and recent recombination are investigated to identify genomic regions that remain distinct between species and those that do not, suggesting possible erosion of barriers to gene flow between the two species. Finally, we discuss the possibility that realized recombination could be linked to a recent ecological overlap in the human niche. Our study offers a complementary approach to functional genome analyses, making use of the increased availability of large population genomic data sets to better understand the influence of the population structure and ecology on clinical staphylococci.

## Materials and Methods

### Isolates and Sequencing

A total of 83 *S. epidermidis* isolates were selected, from existing archive collections, to represent known diversity within the species and multiple locations and sources (Miragaia et al. 2007; Rolo et al. 2012). These were augmented with 241 *S. aureus* and *S. epidermidis* genomes from public databases, including reference genomes from *S. aureus* strain MRSA252 (Holden et al. 2004) and *S. epidermidis* strain RP62A (Gill et al. 2005), to give a total of 324 isolate genomes. There are limitations associated with using data sets containing published genomes from multiple studies, such as the lack of control on sampling and isolate information, but these genomes, principally from clinical isolates, are from diverse lineages (Miragaia et al. 2007). The majority of the *S. aureus* isolates were from clinical infection (*n* = 168) and *S. epidermidis* isolates were from clinical infections (*n* = 67) and asymptomatic colonization (*n* = 72) (supplementary table S1, Supplementary Material online). Almost all isolates from both species originated from humans, with the exception of 7 *S. aureus* isolates from poultry and ruminants (supplementary table S1, Supplementary Material online).

For genomes sequenced in this study, DNA was extracted using the QIAamp DNA Mini Kit (QIAGEN, Crawley, UK), using manufacturer’s instructions with 1.5 μg/μl lysostaphin (Ambi Products LLC, NY) to facilitate cell lysis. DNA was quantified using a Nanodrop spectrophotometer, as well as the Quant-iT DNA Assay Kit (Life Technologies, Paisley, UK) before sequencing. High-throughput genome sequencing was performed using a HiSeq 2500 machine (Illumina, San Diego, CA), and the 100 bp short read paired-end data was assembled using the de novo assembly algorithm, Velvet (Zerbino and Birney 2008; version 1.2.08). The VelvetOptimiser script (version 2.2.4) was run for all odd k-mer values from 21 to 99. The minimum output contig size set to 200 bp with the scaffolding option switched off; all other program settings were left unchanged. An average of 413 million nucleotides were processed in 44 min with Velvet running in parallel using 12 threads on a 64-core Dell PowerEdge R815 Server. Apart from one genome (isolate SS_0376; assembled in 1,146 contigs for a total assembled sequence size of 3,230,689 bp), the average number of contigs in 82 newly sequenced *S. epidermidis* genomes was 75.9 for an average total assembled sequence size of 2,518,294 bp (supplementary table S6, Supplementary Material online).

### Core and Accessory Genome Variation

A reference pan-genome approach (Meric et al. 2014) with gene-by-gene alignment and whole-genome MLST (Jolley and Maiden 2010; Sheppard et al. 2012; Maiden et al. 2013) was implemented using BIGSdb open source software (Jolley and Maiden 2010). First, a reference gene list was assembled from two publicly available genomes, *S. aureus* strain MRSA252 (Holden et al. 2004; GenBank: NC_002952) and *S. epidermidis* strain RP62A (Gill et al. 2005; GenBank: NC_002976). The total number of genes in these isolates was 5,496 and after removal of 2,010 duplicate genes, that were present in both genomes with >70% nt identity across ≥10% of the gene, the reference pan-genome contained 3,486 loci. Second, loci in the 324 genomes of all isolates were identified by using BLAST comparison to this list with a >70% nt sequence identity on ≥50% of sequence sufficient to call a locus match. Consistent with whole-genome MLST (Meric et al. 2014), a matrix was produced summarizing the presence/absence, allelic diversity, and possible homologs in both species of reference pan-genome genes, based upon these BLAST parameters. The proportions of genes at each locus that were missing or incomplete were calculated for *S. aureus* and *S. epidermidis*. For each pair of isolates, the number of shared genes and alleles (identical sequences at a given locus) was calculated and the core genome for each species, and for the genus, was defined as the complement of genes that were present in all isolates. Concatenated gene sequence files were submitted to RAST, an automated annotation pipeline (Aziz et al. 2008). The output contained a functional classification and description of the predicted gene product for each gene obtained from the SEED database (Devoid et al. 2013; Overbeek et al. 2014). The quality of genome assemblies did not impact the detection, using BLAST, of specific virulence genes (supplementary fig. S4, Supplementary Material online). Indeed, most of the allelic variation was accurately characterized using our approach, as the average number of contigs for the assemblies was 78.17, corresponding to an average contig size of 56,035.12 bp (average N_95_ of 32,949.02 bp) (supplementary table S6, Supplementary Material online). A typical gene of *Staphylococcus* sp. is around 1 kb in length, which made incomplete genes rare in our analysis. Furthermore, when estimating the core genome, incomplete genes were considered to be present to account for technical artifacts associated with fragmented genomes.

### Estimating Core Genome Nucleotide Diversity

To infer nucleotide diversity, we used 1,225 core genes shared by the two species without truncation in any of the 324 genome used, which could be caused by the gene being present at the end of an assembled contiguous sequence. For each of these core genes, we calculated the Watterson’s estimator of population mutation rate (Watterson 1975) and Tajima’s *D* (Tajima 1989) using DnaSAM (Eckert et al. 2010). The overall distribution of estimators for each gene and each species were compared using a Mann–Whitney *U* test (Wilcoxon’s rank sum test).

### Estimating Genealogies

Trees were constructed based on 2,059 core *S. aureus* loci or 2,058 core *S. epidermidis* loci, and 1,478 loci found in the genomes of isolates of both species. Genes were aligned individually using MUSCLE (Edgar 2004) and concatenated to produce contiguous sequence alignments. A recompiled version of FastTree 2.1.7 (Price et al. 2010) was used to reconstruct an approximation of a maximum-likelihood tree with branch lengths greater than 0.0000005, which corresponds to a minimum branch length of 1 substitution for every 2,000,000 bp (1,000 times more than the default FastTree parameters). The software was run with the –gtr option to use a generalized time-reversible model of nucleotide evolution, and trees were visualized and annotated using MEGA6 (Tamura et al. 2013). Individual gene trees were constructed in the same way to examine their distribution between species.

### Pairwise Genome Comparison

Core genome nucleotide diversity was examined between representative isolates of *S. aureus* and *S. epidermidis*. Nucleotide identity in the core genome was calculated for each pair of genomes using the “percentage_identity” BioPerl module (fig. 2*B*). Pairwise comparisons between isolates were averaged to give an estimate within and between species. Specifically, core genome nucleotide divergence within *S. aureus* was averaged from the comparison of the following pairs of isolates: WW2703/97—ST398; WW2703/97—MW2; 21310—A9719; ATCC BAA-39—O46. Core genome nucleotide divergence within the *S. epidermidis* genomes was averaged from the comparison of the following pairs of isolates: NIH04008—14.1.R1.SE; VCU071—NIH08001; VCU071—NIH04008. Core genome nucleotide divergence between *S. aureus* and *S. epidermidis* was averaged from the comparison of the following pairs of isolates: WW2703/97—14.1.R1.SE and NIH08001—ST398.

### Intraspecific Core Genome Recombination

We used the BratNextGen (BNG) software (Marttinen et al. 2012) to estimate the amount of homologous recombination in the core genome of *S. aureus* and *S. epidermidis* and to obtain recombination-free input sequences for phylogenetic analyses. Analyses of 181 *S. aureus* genomes and 143 *S. epidermidis* genomes were conducted separately. The BNG input alignment files contained 232,780 single nucleotide polymorphisms (SNPs) for the 181 *S. aureus* isolates, and 123,338 SNPs for the 143 *S. epidermidis* isolates. Proportion of shared ancestry (PSA) tree cutoffs equal to 0.06 and 0.11 were used for *S. aureus* and *S. epidermidis*, respectively. Both values were supported by a clear clustering pattern in the PSA trees. A total of 20 iterations of hidden Markov model (HMM) parameter estimation were performed for both alignments and BNG detected 26 groups of *S. aureus* isolates and 56 groups of *S. epidermidis* isolates. Statistically significant (*P* value not exceeding 5%) recombination in the core genome was determined with 100 parallel permutation runs executed on a cluster computer. The negligible changes (difference smaller than 0.001 between subsequent iterations) in HMM parameter values observed after 50% of the iterations indicated sufficient convergence in the estimation procedure. To establish genetically distinct groups of strains at a genome-wide level, hierarchical BAPS clustering (Cheng et al. 2013) was performed with the same input alignments as for BNG using default settings with 50–75 clusters as the a priori upper bound and two nested levels. Exactly identical clustering results were obtained in ten separate runs of the software. Recombination-free alignments were created by masking all recombinant segments as missing data in the core genome alignments of 181 and 143 *S. aureus* and *S. epidermidis* genomes, respectively. The length of inferred recombinant fragments for each species was also examined and presented in the text.

### Interspecific Accessory Genome Recombination

A tree-based method was used for detecting recombination between species (de Been et al. 2013). Briefly, in the absence of recombination, a bifurcating tree is expected where genes are grouped into species clusters. Where a gene sequence clusters with those from the other species, interspecies recombination is inferred (Sheppard et al. 2008). Gene-by-gene alignments were produced for all genes using MUSCLE (Edgar 2004). Accessory gene alignments were produced, excluding 1,478 genes shared by all isolates (fig. 2), and 745 alignments contained at least one sequence from both species. After filtering out eight gene alignments that did not contain at least one variable position, phylogenetic trees for the remaining 737 gene alignments were built using RAxML under the general time reversible substitution model (Stamatakis 2014). All resulting trees were midpoint rooted using the Phangorn package in R software (Schliep 2011). We then checked the congruence of the rooted trees with an expectation of complete differentiation between *S. aureus* and *S. epidermidis* at the first major phylogenetic split—closest to the root. Four hundred out of 737 gene trees displayed a clear phylogenetic split into *S. aureus* and *S. epidermidis* clades, with no mixture between species. For 137 genes where there were at least 100 variable sites, the two branches were associated with a species based on majority rule. The degree of species overlap was derived from the number of genes of one species nested within the branch where the majority were from the other species. To avoid spuriously large or small values of overlap due to genes that were rarely present, the overlap was calculated only using genes present in at least 30% of the isolates of both species, leaving 62 gene trees. The distributions of the fractions of strains nested in the other species were compared between *S. aureus* and *S. epidermidis* with the nonparametric Kolmogorov–Smirnov test assuming a null equal distribution. There was no evidence of asymmetric connectivity between species (*P* = 0.45) and directionality of the connectivity remained unresolved.

## Results

### Whole-genome MLST

**Fig. 1.—:**
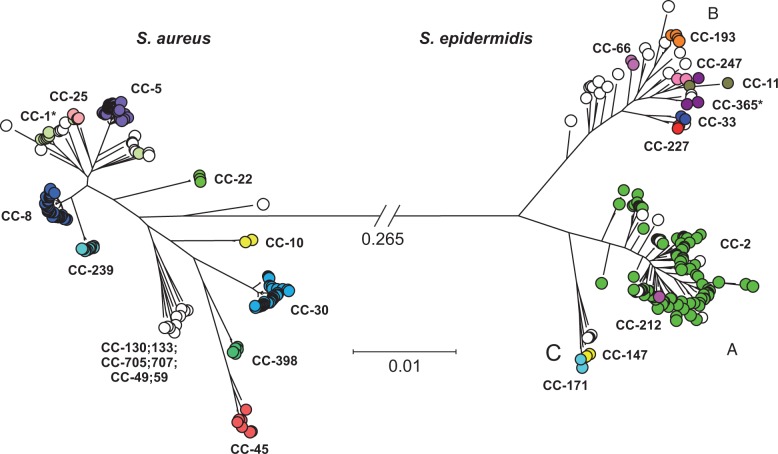
Population structure of 181 *S. aureus* and 143 *S. epidermidis* isolates. A phylogenetic tree constructed from 1,478 genes (2,066,448 bp) found in both species using an approximation of the maximum-likelihood algorithm, implemented in FastTree2. The scale represents the number of substitutions per site. The branch linking *S. aureus* (left) to *S. epidermidis* (right) is 0.265 substitutions per site long, corresponding to 537,276 variable sites. Isolates are colored according to clonal complexes and asterisks denote clonal complexes that appear paraphyletic on this tree, including the *S. aureus* CC-1 and the *S. epidermidis* CC-365.

MLST, based upon seven loci, has been successfully used for epidemiological surveillance and evolutionary studies of staphylococci. Here, we extend this method to the whole genome by identifying a core set of 1,478 genes universally present within the 181 *S. aureus* isolates and 143 *S. epidermidis* isolates in this study. The *S. aureus* tree (fig. 1) revealed distinct clusters which closely match the clonal complexes definitions inferred by eBURST (Feil et al. 2004) analysis of the *S. aureus* MLST database (Feil et al. 2003) (as of December 2013). Of 181 isolates in our data set, 116 belonged to three major lineages: CC-5 (*n* = 54); CC-8 (*n* = 41, including 9 CC-239 isolates); CC-30 (*n* = 30). Other clonal complexes were represented by fewer strains: CC-45 (*n* = 6); CC-398 (*n* = 5); CC-22 (*n* = 3); other complexes (*n* = 16).

In contrast to the highly structured clonal lineages in the *S. aureus* data set, fewer discrete clusters were evident in the *S. epidermidis* tree, and there was less consistency with clonal complexes defined using MLST and eBURST (fig. 1; Feil et al. 2004). For example, CC-212 is nested within CC-2 rather than forming a distinct cluster. Other clonal complexes were also represented clustering into two further groups. Group B was diverse and included isolates belonging to the CC-193, CC-365, CC-11, CC-33, CC-66, CC-212, CC-227, CC-247 clonal complexes, all with <5 isolates (supplementary table S1, Supplementary Material online, which also includes a full list of individual sequence types). Group C was a smaller cluster related to group A, and included isolates belonging to the CC-147 and CC-171 complexes. This observed clustering of isolates is consistent with a previous study based on 129 *S. epidermidis* isolates characterized by MLST, with MLST groups GC1, GC5, and GC6 being included in our group A, GC2 and GC4 our group B, and MLST group GC3 our group C (Thomas et al. 2014). The 83 new genomes added in this study increased representation of group B and C isolates (supplementary fig. S1, Supplementary Material online). Isolate information, including genome sequence files are publically available (Dryad, doi:10.5061/dryad.82jq4).

### Core and Accessory Genome Variation

**Fig. 2.—:**
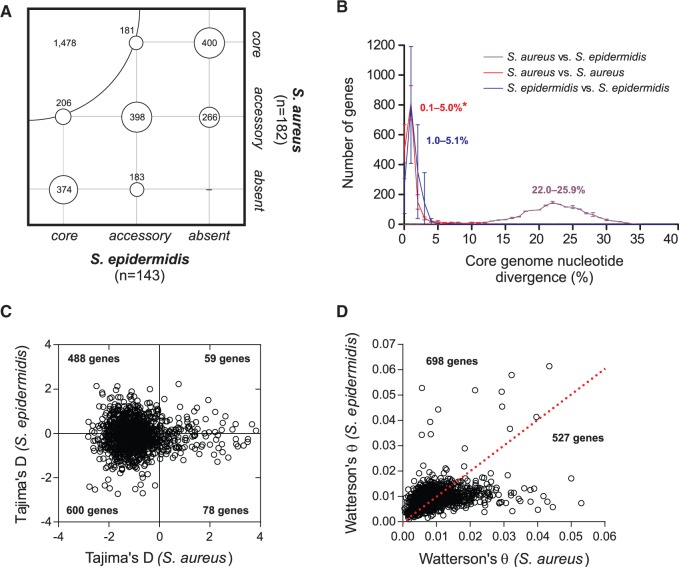
Core and accessory genome variation in *S. aureus* and *S. epidermidis*. (*A*) Overlap between the core and accessory genomes calculated in 324 *S. aureus* and *S. epidermidis* genomes. Core genes were defined as being present in 100% isolates, accessory genes in less than 100% but more than 0%, and absent in 0%. The radius of each circle is proportional to the number of detected genes. (*B*) Core genome nucleotide divergence of representative pairs of *S. aureus* and *S. epidermidis* strains between each other. The numbers indicate the range of calculated nucleotide divergence for at least three pairs of strains, and the error bars indicate standard error of the mean. (*C*) Tajima’s *D* values for 1,225 core genes shared by both species. The number of genes with different combinations of positive and negative *D* values is indicated in each quadrant. (*D*) Watterson’s estimator values for each 1,225 core gene shared by both species. More genes showed a higher θ value in *S. epidermidis* than in *S. aureus*.

We defined core gene sets as those genes common to all isolates, both at the level of individual species (species core), and by considering both species combined. Regarding the latter, we identified 1,478 genes that were universally present in all 181 *S. aureus* and 143 *S. epidermidis* isolates (fig. 2*A*) corresponding to approximately 52% of the full gene complement of an *S. aureus* genome, and 56% of the full gene complement of an *S. epidermidis* genome, based on average gene content. At the individual species level, a total of 2,059 genes were shared by all *S. aureus* isolates in this study and 2,058 by all *S. epidermidis* isolates, corresponding to a species core genome sensu stricto of approximately 73% and 78% of all genes for *S. aureus* and *S. epidermidis* isolates in this study (fig. 2*A*). Our results were consistent withearlier reports of core genome size, with 2,001 shared genes in 16 genomes of *S. aureus* (Suzuki et al. 2012) and 1,960 shared genes in 30 genomes of *S. epidermidis* (Conlan et al. 2012). Moving from an intra- to interspecies comparison therefore resulted in the loss of approximately 500 genes from the core gene set. This decrease in the core gene set was surprisingly small given the strikingly different timescales between intra- and interspecies divergences. To illustrate this, the pairwise nucleotide divergence of core genes ranged from 0.1% to 5% within species, but from 22.0% to 25.9% between species (fig. 2*B*). The conservation of the core gene set thus essentially extends to both species. This observation contrasts markedly with other species with “open” pan-genomes such as *Escherichia coli* (Touchon et al. 2009). Although it is well known that closely related *S. aureus* strains (i.e., belonging to the same clonal complex) can differ in gene content, these differences are largely due to a relatively small number of hypermobile phage and other dynamic elements. Our analysis suggests that the rate of gene content change in staphylococci slows rapidly and substantially over greater divergence times and that approximately half of any given *S. aureus* or *S. epidermidis* genome has been universally retained in at least these two species.

This work, and the identification of a set of 1,478 core staphylococci genes (supplementary file S1, Supplementary Material online), provides a convenient and comprehensive tool for understanding genome-wide relationships in staphylococci, which is computationally feasible and which captures genome-wide relationships at a fine enough scale for within-species comparisons while retaining the ability to resolve interspecies gene replacements. The *S. aureus*- and *S. epidermidis*-specific core genomes, those genes universally present in one species and universally absent in the other, was 374 for *S. epidermidis* and 400 for *S. aureus* isolates in this study. A total of 1,234 genes comprised the accessory genome, that is, they were absent in at least one *S. aureus* or *S. epidermidis* isolate (fig. 2*A*). The average *S. epidermidis* core gene had 0.182 ± 0.077 unique alleles per isolate, compared with 0.159 ± 0.061 for *S. aureus.* The value for *S. epidermidis* was significantly greater, based upon a two-tailed Mann–Whitney *U* test (*P* < 0.0001). Additionally, we calculated Tajima’s *D* (Tajima 1989; fig. 2*C*) and the genetic diversity estimator Watterson’s θ (fig. 2*D*). There were 600 and 59 core genes with negative and positive values of Tajima’s *D* in *S. epidermidis* and *S. aureus*, respectively (fig. 2*C*). Notably, there were 488 core genes with a positive value of Tajima’s *D* in *S. epidermidis* but a negative value in *S. aureus*, indicating that they varied in their allelic diversity and frequency, with a higher tendency for multiple alleles to be present at varying frequencies in *S. epidermidis*, whereas rare alleles of the same genes were present at low-frequency in *S. aureus* (fig. 2*C*). Conversely, only 78 genes showed a positive value of Tajima’s *D* in *S. aureus* and negative value in *S. epidermidis* (fig. 2*C*). The overall distribution of *D* values was statistically different between the two species (Mann–Whitney *U* test, *U* = 277,296, *P* < 0.0001). Additionally, we calculated Watterson’s θ (Watterson 1975) as a way to estimate the population mutation rate in the core genome of the two species (fig. 2*D*). The distribution of θ values was significantly different between the species (Mann–Whitney *U* test, *U* = 688,972, *P* = 0.0005), with around 700 core genes showing a higher θ for *S. epidermidis* than *S. aureus*, which is indicative of a higher genetic polymorphism (fig. 2*D*). However, these results are highly sample dependent so general inference is not made from these results alone.

**Fig. 3.—:**
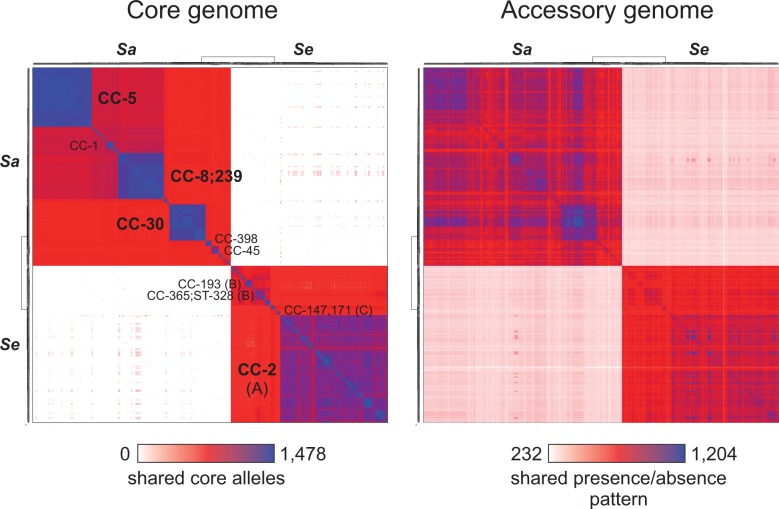
Pairwise core and accessory genome variation in *S. aureus* and *S. epidermidis*. Matrices show pairwise comparison between 324 isolates ordered according to the phylogenetic tree presented in figure 1. Core genome similarity is based upon the number of shared alleles at 1,478 loci found in all isolates. Accessory genome similarity based upon gene presence or absence at 2,008 noncore loci. The heat-map coloring ranges from white, through red, to blue (maximum). The minimum number of shared alleles in the core genome was 0 and the maximum was 1,478. The minimum number of shared accessory genes was 232 and the maximum was 1,204.

In our approach, each unique allele was assigned an identifier, and differences between isolates are thus scored on the basis of the number of allelic mismatches. We examined the validity of this simple clustering strategy by comparing the patterns of relatedness based on a full phylogenetic approach with those based on allelic mismatches (fig. 3). There were three major clusters of core genome similarity in *S. aureus*, corresponding to the main clonal complexes resolved by phylogeny (CC-5, CC-8, and CC-30), hence these approaches are consistent at this level. This was also true, but to a lesser extent, in *S. epidermidis*, where groups A, B, and C showed higher core gene allelic similarity within the groups than between them (fig. 3*A*), and some subclustering was observed within the ST-2 complex.

Lindsay et al. (2006) noted that each clonal complex in *S. aureus* also contains a distinct repertoire of accessory genes. Conversely, this implies that clonal complexes may have their own core genome; those genes universally present within a given clonal complex but which differ between them are assigned as “core variable” (Lindsay et al. 2006). In order to examine this further, in addition to core gene allelic identity (identical/nonidentical) we also considered to what extent accessory gene content differences (presence/absence) are consistent with the phylogeny. The clusters of strains corresponding to clonal complexes in *S. aureus* support the existence of core variable genes in this species (fig. 3*B*), as suggested by Lindsay et al. (2006). In general, however, accessory gene content differences show less consistency with the phylogeny than core gene allelic identity. In *S. aureus*, groups of isolates within CC-30 and CC-5 shared a larger number of genes with one another than with some more closely related clonal complexes (fig. 3*B*). In *S. epidermidis*, accessory genome content was not strongly clustered within clonal complexes (fig. 3*B*).

We also carried out the analyses of core gene allelic identity (identical/nonidentical) and accessory gene (presence/absence) at an interspecies level. Consistent with the degree of nucleotide divergence between species, relatively few core genome alleles were shared by isolates from the two species, with an average of 1.824 ± 0.982 alleles out of the 1,478 examined genes having identical sequences in isolates from the two species. The maximum number of alleles shared by two isolates from the two species was 9. In contrast, much of the accessory genome was shared between species with on average 325.4 genes found to be present or absent similarly in any given pair of *S. aureus* and *S. epidermidis* genomes (fig. 3*B*). This number represented 16.2% of the 2,008 accessory genes identified in this study (fig. 2*A*), or 12.4% and 11.6% of the average *S. epidermidis* and *S. aureus* genome, respectively.

### Population Structure and Homologous Recombination

**Fig. 4.—:**
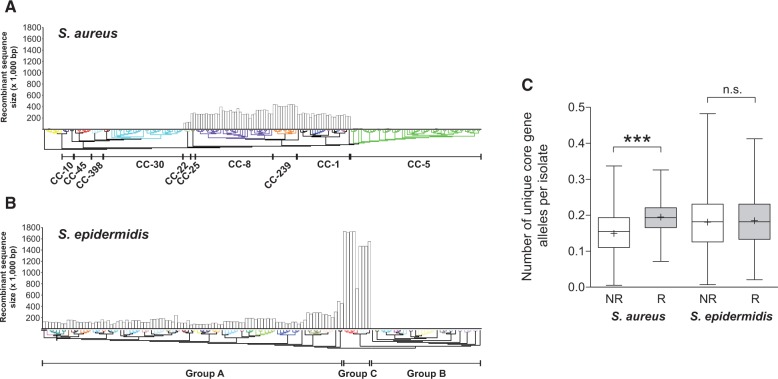
Population structure and core genome recombination in *S. aureus* and *S. epidermidis*. Phylogenetic tree reconstructions from core gene alignments, using an approximation of the maximum-likelihood algorithm, for (*A*) 181 *S. aureus* and (*B*) 143 *S. epidermidis* genomes. Tree branches are colored according to recombining population clusters inferred by BNG and histograms indicate the total length of recombining sequence inferred. (*C*) The distributions of unique alleles of recombining (R; light gray) and nonrecombining (NR; white) core genes. Asterisks indicate a statistical significance of *P* < 0.0001, n.s. denotes no significance.

To better understand the evolutionary history of separation of *S. aureus* and *S. epidermidis* lineages, we estimated the number of ancestral populations by grouping isolates into genetically divergent clusters using BNG (Marttinen et al. 2012). The algorithm inferred the positions and sizes of DNA sequence segments with evidence of homologous recombination and grouped isolates according to recombination pools (fig. 4). There were 26 *S. aureus* sequence clusters, 17 of which contained two or more isolates. There were 56 *S. epidermidis* sequence clusters, 32 of which contained more than two isolates. Homologous recombination was unevenly detected across *S. aureus* and *S. epidermidis* sequence clusters (fig. 4). The sequence cluster most affected by recombination in *S. aureus* corresponds to CC-239. This is expected as this lineage is known to have emerged via a single very large homologous replacement affecting approximately 20% of the genome (Robinson and Enright 2004). CC-8, CC-1, and CC-25 have also been affected by recombination. These clonal complexes belong to the same major clade within the *S. aureus* tree, which has been recently demonstrated to have experienced higher levels of recombination than other branches (Everitt et al. 2014). However, CC-5 also belongs to the same clade, and this appears to have recombined less. This observation is different from an earlier study (Everitt et al. 2014). For *S. epidermidis*, recombination occurs mainly in groups A and C. There is little evidence for recombination within group B, which confirms observations from an earlier MLST study (Thomas et al. 2014). The observation of a frameshift in *dprA* in 11 CC-5 vancomycin-resistant *S. aureus* (VRSA) isolates led a previous study to hypothesize that CC-5 could acquire foreign DNA more easily, as this gene is involved in natural transformation in other bacterial species (Kos et al. 2012). In this study, there was little evidence of recombination in CC-5 (fig. 4) and the *dprA* frameshift was only detected in 42/107 (39.2%) CC-5 isolates from our data set, which include the 12 VRSA isolates mentioned above. This suggests that frameshift mutation in *dprA,* alone, may not be sufficient to promote the acquisition and recombination of DNA.

To examine the effect of the inferred recombination on the population structure of both species, trees were constructed from core genome sequence alignments from *S. aureus* and *S. epidermidis*, respectively, and from the same alignments with inferred recombinant sequences masked by replacing them with gaps. In the case of *S. aureus*, the tree topology remained essentially unchanged which is consistent with the view that recombination has not occurred at a sufficient frequency to compromise the phylogenetic signal in this species. A notable exception is the hybrid lineage CC-239. As expected, this lineage clusters nearer the closely related CC-8 lineage after recombinant sequence has been removed. A further discrepancy is noted with ST-34 and ST-42 which are also a hybrid lineage (Robinson and Enright 2004). After removal of recombination this lineage clusters more closely with the parent lineage CC-30 (supplementary fig. S2, Supplementary Material online). For *S. epidermidis*, the major topological shift resulting from the exclusion of the regions affected by recombination is the closer clustering of the group C isolates (including both CC-147 and CC-171) with the major group A. This is consistent with the results presented above and points to a hybrid origin for group C (fig. 4 and supplementary fig. S2, Supplementary Material online).

In *S. aureus* there were significantly more unique alleles in the recombining regions than in the nonrecombining regions (fig. 4*C*). This is consistent with acquisition from discrete nonoverlapping gene pools in *S. aureus,* compared with more widespread recombination in *S. epidermidis,* but could also be explained by shorter recombination blocks in *S. aureus* that create new alleles, or a less representative sampling of *S. aureus*. Recombination appears to have had a greater effect on allelic diversity in *S. aureus* than in *S. epidermidis*. There was a statistical difference between the two in *S. aureus* (unpaired *t*-test; *t* = 8.95 df = 1,476, *P* < 0.0001) but not *S. epidermidis* (unpaired *t*-test; *t* = 0.786 df = 1,476, *P* = 0.432) (fig. 4*C*), suggesting that recombination does not maintain or promote genetic diversity the same way in the two species. When allelic diversity in recombinant and nonrecombinant regions was compared in isolates from both species, the difference was still highly significant (unpaired *t*-test; *t* = 0.506 df = 1,476, *P* < 0.0001), as were the variances between all distributions (one-way ANOVA, *P* < 0.0001). This could also be explained by the possibility that the recombining regions in *S. aureus* are smaller than in *S. epidermidis*. We examined the lengths of inferred recombinant tracts and observed that the median size of all recombinant tracts was 654 bp for *S. aureus* and 1,010 bp for *S. epidermidis* (supplementary table S5, Supplementary Material online). We calculated the average gene size of a gene shared by the two species to be around 930 bp, which is lower than the recombination tract length for *S. epidermidis*, but higher for *S. aureus*. This observation provides additional support to the hypothesis that recombination in *S. aureus* is more likely than in *S. epidermidis* to generate novel recombinant alleles rather than transferring allelic variants of whole core genes.

### Predicted Functions of Recombining Genes

We assigned putative functional categories to those genes inferred to have experienced homologous recombination with BNG, and nonhomologous recombination inferred with the tree congruence method (de Been et al. 2013). A total of 437 (21.2%) *S. aureus* and 824 (40.0%) *S. epidermidis* core genes showed evidence of homologous recombination. One hundred sixty-five of the 437 *S. aureus* genes (38%) affected by recombination could be assigned to a functional category from the SEED database (Aziz et al. 2008), as were 260 of the 840 genes affected by recombination in *S. epidermidis* (31%). A total of 36 genes showed evidence of homologous recombination in both *S. aureus* and *S. epidermidis,* including genes involved in central metabolism, protein synthesis, and transcription regulation (sigma factor σ_B_; Pane-Farre et al. 2006) (supplementary table S2, Supplementary Material online), but the proportion of recombining genes in the different functional categories was different between species (χ^2 ^= 60.09, df = 16; *P* < 0.0001). Some genes showed evidence of homologous recombination in *S. aureus* or in *S. epidermidis* only (supplementary tables S3 and S4, Supplementary Material online). In *S. aureus,* most (63/102; 62%) of these belonged to just four functional categories (supplementary table S3, Supplementary Material online) and included a putative ferrous iron transporter component (*efeB*)—important in cell-wall structuring and for colonization of the nasal passageway (Weidenmaier et al. 2008) and several genes predicted to encode dehydrogenases involved in fermentation (supplementary table S3, Supplementary Material online). Genes that showed more evidence of homologous recombination in *S. epidermidis* than in *S. aureus* included genes putatively involved in dormancy, arsenic and fosfomycin resistance, and cell wall and capsular synthesis—including those involved in the synthesis and physiology of the capsule (synthesis of sialic acid, mureins involved in peptidoglycan degradation; Vimr et al. 2004) (supplementary table S4, Supplementary Material online). Putative dormancy associated genes, SE_2281 and SE_2285, were respectively annotated as “protein of unknown function identified by role in sporulation (SpoVG)” and “peptidyl-tRNA hydrolase (EC 3.1.1.29)” in the SEED database (Devoid et al. 2013). The presence of homologues does not confirm that these genes were functional.

**Fig. 5.—:**
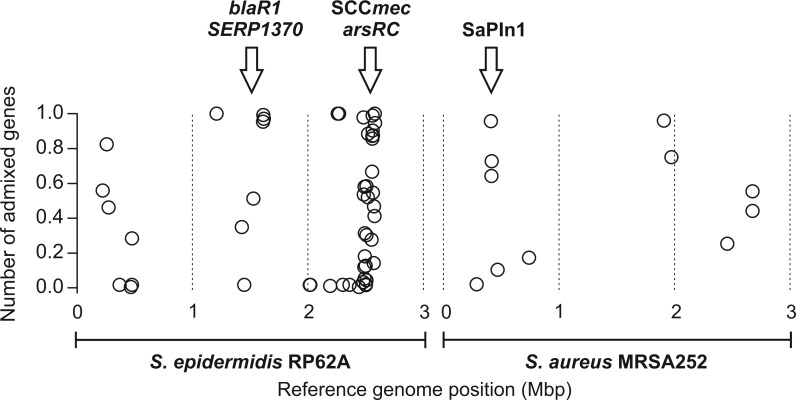
Genomic position of recombined accessory genes in *S. aureus* and *S. epidermidis* based upon single-gene phylogenies. The frequency of admixed genes for 62 accessory genes with mixed ancestry phylogenies mapped to the corresponding locus in the reference *S. epidermidis* (RP62A) and *S. aureus* (MRSA252) genome. Example gene groups with high levels of interspecies gene flow are indicated.

Analysis of nonhomologous recombination between species was carried out using a tree incongruence method (de Been et al. 2013). After filtering single accessory-gene genealogies to remove genes that segregated strongly by species and those where species clusters could not be identified through majority rule (de Been et al. 2013), there were 62 genealogies with a high level of phylogenetic overlap between the two species. Known mobile genetic elements were overrepresented in the resulting gene list, including SCC*mec* genes and associated hypothetical proteins, and the pathogenicity island SaPIn1 (table 1 and fig. 5; supplementary fig. S5, Supplementary Material online). Several genes of SaPIn1 were found to be absent in all *S. epidermidis* (supplementary fig. S5, Supplementary Material online). In addition, transposases, mobile genetic elements, and genes involved in the detoxification and resistance of metals (such as arsenic, cadmium, cobalt, and zinc) also showed evidence of recent genetic exchange between species. This finding was also consistent with an earlier report that metal resistance is affected by recombination in *Staphylococcus* (Chan et al. 2011). By mapping the 62 recombining accessory genes to reference *S. epidermidis* (RP62A) and *S. aureus* (MRSA252) strains, it was possible to see the genomic position of the laterally transferred genes and estimate the degree of admixture (fig. 5). Consistent with the sharing of adaptive genome islands described in previous studies (Gill et al. 2005), single-gene genealogies revealed relatively recent admixture between species, with *S. aureus* and *S. epidermidis* genes clustered on the same, or closely related, branches (supplementary fig. S3, Supplementary Material online).

**Table 1:** Genes for Which Single-gene Phylogenies do not Segregate Clearly by Species (*S. aureus* / *S. epidermidis*)

Gene Name	Alias	Homologs in Other Annotated Genomes^a^	Description	Prevalence in *S. aureus* (*n* = 181)	Prevalence in *S. epidermidis* (*n* = 143)	Frequency of Species Overlap	*S. aureus* Reference Genomic Position^b^	*S. epidermidis* Reference Genomic Position^c^
SERP1173	*—*		IS200 family transposase	60	143	1.000	*—*	1,208,761
SERP2221	*—*		cadmium resistance family protein	75	143	1.000	*—*	2,258,410
SERP2240	*—*		IS200 family transposase	60	143	1.000	*—*	2,268,628
SERP2521	*mecA*		Methicillin resistance penicillin-binding protein MecA	110	74	1.000	*—*	2,578,662
SERP1583	*—*		Mobile element protein	138	136	0.993	*—*	1,612,684
SERP2501	*—*	SA0056, SE0055	hypothetical protein	98	103	0.990	*—*	2,561,164
SERP2422	*—*		FIG01108158: hypothetical protein	181	142	0.979	*—*	2,482,195
SERP1588	*—*		Mobile element protein	139	139	0.971	*—*	1,617,610
SAR1828	*tnpR*	SH1761 (*S. haemolyticus*)	DNA-invertase transposon Tn552	121	75	0.960	1,911,329	*—*
SAR0374	*—*	SA1829, SACOL0896	Hypothetical SAV0790 homolog in superantigen-encoding pathogenicity islands SaPI	84	47	0.957	414,760	*—*
SERP1579	*—*		Mobile element protein	137	131	0.954	*—*	1,610,300
SERP2526	*—*		Mobile element protein	137	131	0.947	*—*	2,581,912
SERP2499	*ccrB*		Cassette chromosome recombinase B	108	93	0.903	*—*	2,560,296
SERP2467	*—*		FIG01108090: hypothetical protein	113	123	0.885	*—*	2,521,017
SERP2504	*—*	SA0054, SE0030	FIG01108090: hypothetical protein	113	123	0.876	*—*	2,562,086
SERP2496	*—*	SA0059	hypothetical protein	107	86	0.872	*—*	2,557,063
SERP2498	*ccrA*		Cassette chromosome recombinase A	108	84	0.857	*—*	2,558,646
SERP0249	*—*	SA0026, SA0034, SE0071, SE0079, SE0090	IS431mec-like transposase	137	131	0.825	*—*	257,823
SAR1893	*—*	SACOL1857	hypothetical protein [Genomic island νSaβ2]	110	44	0.750	1,977,960	*—*
SAR0382	*—*	SA1820, SAV0800	Putative terminase, superantigen-encoding pathogenicity islands SaPI	121	92	0.728	421,037	*—*
SERP2495	*—*	SA0060	FIG01108228: hypothetical protein	72	48	0.667	*—*	2,555,270
SAR0377	*—*	SA1826, SACOL0900	Hypothetical SAV0794 homolog in superantigen-encoding pathogenicity islands SaPI	91	56	0.643	417,811	*—*
SERP2453	*—*		hypothetical protein	181	142	0.585	*—*	2,506,887
SERP2431	*arsC-2*	SAR0692	Arsenate reductase (EC 1.20.4.1)	105	139	0.581	*—*	2,489,753
SERP0209	*arsC-1*	SAR0692	Arsenate reductase (EC 1.20.4.1)	105	143	0.559	*—*	221,772
SAR2594	*—*	SE0212, SA2302	ABC transporter ATP-binding protein	181	74	0.554	2,679,952	*—*
SERP2503	*—*	SA0057	FIG01107894: hypothetical protein	108	108	0.546	*—*	2,561,562
SERP2423	*—*		FIG01108158: hypothetical protein	181	142	0.536	*—*	2,483,315
SERP2465	*—*		lipoprotein	181	142	0.521	*—*	2,519,564
SERP1460	*blaR1-1*		Beta-lactamase regulatory sensor-transducer BlaR1	132	115	0.513	*—*	1,526,822
SERP2515	*—*	SA0044	Disulfide bond regulator	124	111	0.468	*—*	2,571,330
SERP0265	*—*		Mobile element protein	173	142	0.462	*—*	272,732
SAR2595	*—*	SA2303, SE0213	Membrane spanning protein	181	61	0.443	2,680,724	*—*
SERP2520	*mecR1*		Methicillin resistance regulatory sensor-transducer MecR1	104	69	0.413	*—*	2,576,556
SERP1370	*—*		Cadmium resistance protein	75	143	0.350	*—*	1,428,085
SERP2434	*—*	SA0046	Disulfide bond regulator	124	111	0.315	*—*	2,492,038
SERP2452	*—*		Conserved domain protein	62	79	0.304	*—*	2,506,105
SERP0479	*—*		Mobile element protein	162	143	0.284	*—*	476,253
SERP2491	*—*	SA1014	hypothetical protein	181	141	0.276	*—*	2,551,832
SAR2392	*—*	SA2101	Hypothetical protein	181	142	0.254	2,461,526	*—*
SERP2435	*—*	SA0043	Zn-dependent hydroxyacylglutathione hydrolase / Polysulfide binding protein	127	110	0.181	*—*	2,493,385
SAR0698	*tnp*	SACOL0036, SE0629	Putative transposase	138	109	0.174	743,744	*—*
SERP2513	*—*	SA0046	FIG003846: hypothetical protein	124	112	0.143	*—*	2,569,855
SERP2443	*—*		FIG01110589: hypothetical protein	181	142	0.127	*—*	2,500,392
SERP2429	*arsR-2*	SA1591, SAR0690, SE0136	Arsenical resistance operon repressor	181	134	0.119	*—*	2,488,047
SAR0445	*lpl5*	SA0401	Tandem lipoprotein within Pathogenicity island	173	99	0.104	473,389	*—*
SERP2432	*—*	SA0080	FIG003846: hypothetical protein	124	112	0.054	*—*	2,490,572
SERP2451	*—*		Tandem lipoprotein within Pathogenicity island	181	139	0.044	*—*	2,505,694
SERP2418	*—*		FIG01109629: hypothetical protein	180	128	0.033	*—*	2,479,100
SAR0248	*—*	SA0243, SA0247, SE0321, SERP0198	Teichoic acid biosynthesis protein B	179	142	0.021	290,744	*—*
SERP2445	*—*		FIG01108771: hypothetical protein	181	142	0.021	*—*	2,501,497
SERP0369	*—*		Mobile element protein	176	143	0.017	*—*	367,885
SERP0478	*—*		Mobile element protein	176	143	0.017	*—*	476,113
SERP1393	*—*		Mobile element protein	176	143	0.017	*—*	1,449,378
SERP1995	*—*		Mobile element protein	176	143	0.017	*—*	2,014,786
SERP2002	*—*		Mobile element protein	176	143	0.017	*—*	2,020,995
SERP2268	*—*		Mobile element protein	176	143	0.017	*—*	2,301,670
SERP2323	*—*		Mobile element protein	176	143	0.017	*—*	2,360,017
SERP2447	*—*		FIG01108158: hypothetical protein	181	142	0.017	*—*	2,502,826
SERP2163	*—*		Mobile element protein	177	143	0.011	*—*	2,192,271
SERP2390	*sspB*		Staphopain A precursor (EC 3.4.22.48)	180	141	0.006	*—*	2,441,865
SERP0465	*—*		Cobalt–zinc–cadmium resistance protein	181	142	0.006	—	467,605

^a^As inferred from the AureusDB website: http://aureusdb.biologie.uni-greifswald.de/ (last accessed April 22, 2015).

^b^Position on the reference genome of *S. aureus* strain MRSA252 [GenBank accession: NC_002952].

^c^Position on the reference genome of *S. epidermidis* strain RP62A [GenBank accession: NC_002976].

It should be noted that the single-gene trees from SCC*mec* elements were incongruent with the whole-genome phylogeny which robustly separates the two species (supplementary fig. S3, Supplementary Material online). This is consistent with the high mobility of SCC*mec* elements between *S. aureus* and *S. epidermidis* (Forbes and Schaberg 1983; Hanssen et al. 2004; Maslanova et al. 2013).

### Distribution of SCCmec Genes in *S. aureus* and *S. epidermidis*

The distributions of genes and alleles of the SCCmec element (*mecA*, *mecI*, *mecR1*, *ccrA*, and *ccrB*) were analyzed across the full *S. aureus* and *S. epidermidis* data set. The methicillin resistance structural gene *mecA* was present in 215/325 isolates (111/182 *S. aureus,* 74/143 *S. epidermidis*) with 23 unique alleles detected. Allelic variation was higher in *ccrA* (58 alleles) and *ccrB* (55 alleles) and lower in *mecI* (9 alleles) and *mecR1* (14 alleles). *mecA* and *mecR1* were always present together, as expected with known SCC*mec* types (International Working Group on the Classification of Staphylococcal Cassette Chromosome Elements 2009), and *mecI* was detected in 123/215 (57%), consistent with frequencies in published data (International Working Group on the Classification of Staphylococcal Cassette Chromosome Elements 2009). The absence of genes from the *mec* gene complex was not always commensurate with the absence of genes from the *ccr* gene complex and 30/215 isolates were *mecR1*-negative but *ccrB*-positive. As expected, the majority of *mecA*-positive isolates in our sample were isolated from infection, but 27 were from healthy carriage.

## Discussion

The proliferation of large data sets allows us to start to relate population genomics to ecology. An individual genome contains evidence of the niche to which it is adapted, which we can study using the methods of comparative genomics. A population sample extends this analysis by allowing us to compare the patterns of gene sharing, by homologous and nonhomologous recombination. Recombination in bacteria is influenced by physical proximity between lineages. Recombination could be direct—lineages coexist in an ecological niche and physically exchange DNA via conjugation or transformation, or indirect—lineages exchange DNA without direct physical interaction, via transduction, which has been recently shown to occur in *Staphylococcus* (Chlebowicz et al. 2014; Uchiyama et al. 2014). This concept of extended ecological interactions could allow inference of an overlapping element of the niche (Hutchinson 1959) that applies to bacterial pathogens of distinct species sharing the same host. Where recombination occurs among accessory genes influenced by a common selective pressure, enhanced opportunity to recombine resulting from common niche space is likely to increase realized recombination. Here, we have shown that *S. epidermidis* and *S. aureus* isolated mostly from hospitals have shared not only resistance loci, which might have been expected, but also genes involved in pathogenicity and metal toxicity resistance. Our data show high levels of recombination, particularly involving genes that are presumed to be advantageous in a hospital setting (table 1 and supplementary fig. S3, Supplementary Material online). The implication is that these genes themselves promote proliferation of subpopulations of the two named species, and that adaptation occurring in one that is of advantage to the other, can be transferred to it.

Over half of the genes identified in the isolates in this study were shared by all *S. aureus* and *S. epidermidis* isolates and only around 19% and 16% of genes were unique to each species respectively. However, despite these similarities in gene content the average nucleotide identity among homologous sequence was around 76%, which is considerably lower than among genes recognizably shared between *E. coli* and *Salmonella enterica,* which are thought to have diverged around 120 Ma (Ochman and Groisman 1994). Some level of gene acquisition and recombination is possible between *S. aureus* and *S. epidermidis* (Wisplinghoff et al. 2003; Hurdle et al. 2005; Diep et al. 2006; Holden et al. 2010)*,* but genetic differentiation can be maintained among bacteria that are frequently sampled from the same location on human skin and nasal pharynx (Frank et al. 2010; Grice and Segre 2011). This could potentially indicate ecological differentiation. An alternative explanation would be that isolates sharing the same niche are under selection for the maintenance of niche-improving genes (Fraser et al. 2009).

Contrasting the genealogies of *S. aureus* and *S. epidermidis* can provide information about differences between these species in terms of genetic diversity. The core genome nucleotide diversity, as inferred by the calculation of Tajima’s *D* and Watterson’s θ estimators (fig. 2*C* and *D*), was significantly different for the two species. Consistently, the tree topology was strikingly different (fig. 1). In *S. aureus,* 80% of isolates clustered into ten discrete clonal complexes while the *S. epidermidis* genealogy showed a much less clear isolate clustering, consistent with a higher rate of recombination between isolates. In *S. epidermidis,* an estimated 40% of core genome genes showed evidence of recent homologous recombination in at least one lineage compared with 21% in *S. aureus* where mutation is thought to generate much of the genetic variation (Feil et al. 2000, 2003). Evidence that recombining core genes in *S. epidermidis* had comparable genetic diversity to nonrecombining genes was also consistent with widespread recombination across the species, although there was variation in gene flow between lineages*.* This reflects a combination of historical and contemporary patterns of gene flow in the core genome but it is difficult to differentiate single large recombination events from frequent smaller ones.

It has been shown that variation in the fraction of the recombining accessory genome correlates with variation in bacterial ecology (Newton and Bordenstein 2011; Rankin et al. 2011; Wiedenbeck and Cohan 2011; Baltrus 2013). In some cases it is possible to make links between ecology and specific mobile element types such as repeats, transposons plasmids, bacteriophages, and insertion sequences (Frank et al. 2002; Moran and Plague 2004; Gill et al. 2005). Building on this, and applying this reasoning to smaller scales of ecological variation between lineages, the whole-genome MLST approach employed here allows the quantification of homologous and nonhomologous recombination for individual genes across the genome and by investigating putative function of recombining genes, the ecological basis of recombinational barriers can be investigated (Sheppard et al. 2014).

As in previous studies, genes that were laterally transferred between *S. aureus* and *S. epidermidis* were commonly associated with mobile genetic elements, including SCC*mec* (Forbes and Schaberg 1983; Hanssen et al. 2004; Maslanova et al. 2013). Additionally, we found that metal resistance (cadmium, arsenic, zinc) and elements of the pathogenicity island SaPIn1 (Novick and Subedi 2007) were also associated with interspecies gene flow. A combination of ecological and evolutionary processes may influence the genes that are shown to recombine between species. First, the amount of niche overlap and its associated presumed increase in opportunity for recombination, and the regularity with which the *S. aureus* and *S. epidermidis* gene pools meet are likely to dictate the increased or reduced opportunity for genetic exchange. Second, the effective population size can vary between the species and lineages and this affects the availability of donor DNA and the frequency of population bottlenecks. For example, *S. epidermidis* may have more recombined genes than the more clonal *S. aureus* because its ubiquity on the skin may expose it to DNA from a more genetically diverse population. If recombination between *S. aureus* and *S. epidermidis* were simply a function of these processes then recombined genes would be expected to be approximately evenly distributed across the genome. This is not the case and the overrepresentation of genes associated with pathogenicity elements and antimicrobial resistance may suggest an adaptive and/or mechanistic role for these elements. Our results are consistent with an *S. aureus* study reporting the detection of recombination in mobile genetic elements integration sites (Everitt et al. 2014), an observation that we also extend to *S. epidermidis* in this study. It is worth mentioning that the transposases affected by interspecies recombination, in addition to facilitating the horizontal transfer of fitness-enhancing genes, could be themselves selfish genetic elements without obvious associated functional or ecological impact (Berg et al. 1984; Aziz et al. 2010).

The extended period of divergence, and large genetic distance between *S. aureus* and *S. epidermidis,* potentially imposes a mechanistic barrier to recombination due to the homology dependence of recombination and other factors promoting DNA specificity (Eggleston and West 1997; Fraser et al. 2007). This explanation can account for the differentiation between the core genomes, with a maximum of only nine shared alleles between any two isolates, suggesting that even when homologous recombination does occur alleles are unlikely to proliferate in the population, possibly because of adaptive incompatibility. This is not the case for some highly mobile elements in the accessory genome, for example the genetic determinants for methicillin resistance clustered on a mobile SCC*mec* element (Jevons 1961; Hiramatsu et al. 2001). An alternative hypothesis to neutral variation could be that, while most recombinant DNA is purged from the population as it is not beneficial, genes that provide a competitive advantage to the recipient genome will proliferate among the progeny. That SCC*mec*, metal resistance and SaPIn1 pathogenicity island elements are exchanged so extensively between clinical *S. aureus* and *S. epidermidis* isolates suggests that anthropogenic factors, such as antibiotic selective pressure, may affect genes in both species.

Human activities can have a dramatic effect on the habitat of bacteria, changing the adaptive landscape and providing new opportunities for the proliferation of recombinant lineages. As has been shown in acid mine drainage areas (Lo et al. 2007) and intensive farms (Sheppard et al. 2008), modern hospitals represent a novel environment for *S. aureus* and *S. epidermidis,* organisms which are thought to have colonized primates and humans for millions of years (Kloos et al. 1976; Kloos 1980; Sakwinska et al. 2011). On this timescale, the greatly enhanced use of antimicrobials represents a rapid change in selective pressure on bacteria that is met with an equally rapid change in their genomes. With the increasing availability of whole-genome data sets for bacterial populations it will be possible to improve understanding of how changes to adaptive landscapes can influence microbial evolution, potentially providing opportunities for interventions to reduce the threat of the proliferation of resistant lineages.

## Supplementary Material

Supplementary materials figures S1–S5 and tables S1–S6 are available at *Genome Biology and Evolution* online (http://www.gbe.oxfordjournals.org/).

Supplementary Data
