# Supplementary material for: Ecological Overlap and Horizontal Gene Transfer in Staphylococcus aureus and Staphylococcus epidermidis
Source: Genome Biol Evol. 2015 Apr 16;7(5):1313–28. doi: 10.1093/gbe/evv066 (PMC4453061; doi:10.1093/gbe/evv066)
Supplement: Supplementary Data [file supp_7_5_1313__index.html]

Ecological overlap and horizontal gene transfer in Staphylococcus aureus and Staphylococcus epidermidis — Ecological Overlap and Horizontal Gene Transfer in Staphylococcus aureus and Staphylococcus epidermidis — Supplementary Data 

# Ecological Overlap and Horizontal Gene Transfer in *Staphylococcus aureus* and *Staphylococcus epidermidis*

## Supplementary Data

files

**Files in this Data Supplement:**

- Supplementary Data - zip file
