# Supplementary material for: Ecological Overlap and Horizontal Gene Transfer in Staphylococcus aureus and Staphylococcus epidermidis
Source: Genome Biol Evol. 2015 Apr 16;7(5):1313–28. doi: 10.1093/gbe/evv066 (PMC4453061; doi:10.1093/gbe/evv066)
Supplement: Supplementary Data [file supp_evv066_suppl_data.zip › New Microsoft Office Word Document.docx]

**Figure S1. The addition of 83 novel *S. epidermidis* genomes increases representation of the species diversity.** The phylogenetic tree was created using a core genome alignment and an approximation of the maximum-likelihood algorithm implemented in FastTree2. Red circles indicate a genome sequenced in this study, white circles indicate a publicly available genome from the NCBI repository, as accessed in February 2013. The scale indicates the number of substitutions per site.

**Figure S2. Effect of core genome recombination on the population structure of *S. aureus* and *S. epidermidis*.** Core genome recombination was inferred by BratNextGen. The recombinant segments were filtered out of a full core-genome alignment to create non-recombinant core genome alignments. Phylogenetic trees were constructed from *S. aureus* (A) recombinant and (B) non-recombinant, as well as *S. epidermidis* (C) recombinant and (D) non-recombinant core genome alignments were created using an approximation of the maximum-likelihood implemented in FastTree2. The colouring of tree leaves corresponds to different *S. aureus* and *S. epidermidis* BratNextGen populations. Arrows indicate pronounced effects on lineage positions between recombinant and non-recombinant core phylogenies.

**Figure S3. Single-gene phylogenies of example genes with evidence of recent admixture between *S. aureus* and *S. epidermidis*.** Twelve single-gene tree reconstructions from alignments, using an approximation of the maximum-likelihood algorithm, belonged to 3 functional groups: SCC*mec*; metal resistance; SaPIn1. In the absence of interspecies genetic exchange, trees would bifurcate with *S. aureus* alleles (grey) on one side and *S. epidermidis* on the other. Evidence for admixture comes from incomplete separation. The scale indicated for each tree represents the number of substitutions per site.

**Figure S4. Detection of specific genes from the SCC*mec* region in genome assemblies of varying quality**. All genomes used in this study, either finished (n=34) or draft assemblies (n=290) were included. Vertical bars for each gene corresponded to the detection of a local alignment between each genome sequence and a reference sequence for the corresponding gene of more than 70% nucleotide identity on more than 50% of the gene length.

**Figure S5. Genetic context and prevalence of genes from the SaPIn1 pathogenicity island in all genomes from this study**. Prevalence of genes from *SAR0368* to *SAR0385* according to the S. aureus MRSA252 annotation was assessed using BLAST in BIGSdb for all *S. aureus* (n=181) and *S. epidermidis* (n=143) genomes used in this study. Some genes were totally absent in all S. epidermidis isolates, while the maximum observed prevalence was *SAR0382* detected in 67.03% *S. aureus* genomes.

**Table S1. Isolates and genomes.**

**Table S2. Predicted functions of genes found to be recombining in both *S. aureus* and *S. epidermidis*.** The different columns labels and contents were defined by the RAST automatic annotation pipeline.

**Table S3. Predicted functions of genes found to be recombining in *S. aureus* but not *S. epidermidis*.** The different columns labels and contents were defined by the RAST automatic annotation pipeline.

**Table S4. Predicted functions of genes found to be recombining in *S. epidermidis* but not *S. aureus*.** The different columns labels and contents were defined by the RAST automatic annotation pipeline.

**Table S5.** **Length distribution of core genome recombinant tracts inferred by BratNextGen using 181 genomes of *S. aureus* and 143 genomes of *S. epidermidis***

**Table S6**. **Summary of statistics on genome sequence assemblies quality.** The statistics were calculated in BIGSdb.

**Supplementary File 1**. Multi-FASTA file containing 1,478 gene sequences shared by all *S. aureus* and *S. epidermidis* from this study.
