# Supplementary material for: Ecological Overlap and Horizontal Gene Transfer in Staphylococcus aureus and Staphylococcus epidermidis
Source: Genome Biol Evol. 2015 Apr 16;7(5):1313–28. doi: 10.1093/gbe/evv066 (PMC4453061; doi:10.1093/gbe/evv066)
Supplement: Supplementary Data [file supp_evv066_suppl_data.zip › Table S2.pdf]

Table S2. Predicted functions of genes found to be recombining in both *S. aureus* and *S. epidermidis* . The different columns labels and contents were defined by the RAST automatic annotation pipeline.

Category	Sub-category	Sub-system	Role
Amino Acids and Derivatives	Arginine; urea cycle, polyamines	Arginine and Ornithine Degradation	Arginine/ornithine antiporter ArcD
	Lysine, threonine, methionine, and cysteine	Threonine degradation	Threonine dehydratase, catabolic (EC 4.3.1.19)
	Aromatic amino acids and derivatives	Common Pathway For Synthesis of Aromatic Compounds (DAHP synthase to chorismate)	3-dehydroquinate synthase (EC 4.2.3.4)
		Phenylalanine and Tyrosine Branches from Chorismate	Prephenate dehydrogenase (EC 1.3.1.12)
Carbohydrates	Central carbohydrate metabolism	Glycolate, glyoxylate interconversions	Phosphoglycolate phosphatase (EC 3.1.3.18)
	Fermentation	Fermentations: Lactate	L-lactate dehydrogenase (EC 1.1.1.27)
Cell Wall and Capsule	Cell Wall and Capsule - no subcategory	UDP-N-acetylmuramate from Fructose-6-phosphate Biosynthesis	Glucosamine--fructose-6-phosphate aminotransferase [isomerizing] (EC 2.6.1.16)
			Phosphoglucosamine mutase (EC 5.4.2.10)
	Gram-Positive cell wall components	Sortase	LPXTG specific sortase A
DNA Metabolism	DNA repair	2-phosphoglycolate salvage	Phosphoglycolate phosphatase (EC 3.1.3.18)
	DNA replication	DNA replication strays	DNA polymerase III polC-type (EC 2.7.7.7)
Fatty Acids, Lipids, and Isoprenoids	Fatty acids	Fatty acid metabolism cluster	Long-chain-fatty-acid--CoA ligase (EC 6.2.1.3)
			3-ketoacyl-CoA thiolase (EC 2.3.1.16)
	Isoprenoids	Isoprenoinds for Quinones	Undecaprenyl diphosphate synthase (EC 2.5.1.31)
Membrane Transport	Uni- Sym- and Antiporters	Proton-dependent Peptide Transporters	Di/tripeptide permease YjdL
Nucleosides and Nucleotides	Purines	Xanthine Metabolism in Bacteria	Hypoxanthine/guanine permease PbuG
			Xanthine permease
Phages, Prophages, Transposable elements, Plasmids	Pathogenicity islands	Listeria Pathogenicity Island LIPI-1 extended	Zinc metalloproteinase precursor (EC 3.4.24.29)
Protein Metabolism	Protein biosynthesis	tRNA aminoacylation, Met	Methionyl-tRNA synthetase (EC 6.1.1.10)
		Programmed frameshift	programmed frameshift-containing
			Peptide chain release factor 2
	Protein processing and modification	G3E family of P-loop GTPases (metallocenter biosynthesis)	Putative metal chaperone, involved in Zn homeostasis, GTPase of COG0523 family
		Protein deglycation	Ribulosamine/erythrulosamine 3-kinase potentially involved in protein deglycation
		Peptide methionine sulfoxide reductase	Peptide methionine sulfoxide reductase MsrA (EC 1.8.4.11)
			Peptide methionine sulfoxide reductase MsrB (EC 1.8.4.12)
Regulons	Atomic Regulons	ar-431-EC Molybdopterin-guanine dinucleotide biosynthesis	Molybdopterin-guanine dinucleotide biosynthesis protein MobB
RNA Metabolism	RNA processing and modification	RNA pseudouridine syntheses	Similar to ribosomal large subunit pseudouridine synthase D, Bacillus subtilis YjbO type
			tRNA pseudouridine synthase B (EC 4.2.1.70)
		tRNA nucleotidyltransferase	tRNA nucleotidyltransferase (EC 2.7.7.21) (EC 2.7.7.25)
Stress Response	Osmotic stress	Choline and Betaine Uptake and Betaine Biosynthesis	Glycine betaine transporter OpuD
			Osmotically activated L-carnitine/choline ABC transporter, ATP-binding protein OpuCA
	Cold shock	Cold shock, CspA family of proteins	Cold shock protein CspA
	Stress Response - no subcategory	SigmaB stress response regulation	RNA polymerase sigma factor SigB
	Periplasmic Stress	Periplasmic Stress Response	Intramembrane protease RasP/YluC, implicated in cell division based on FtsL cleavage
Virulence, Disease and Defense	Resistance to antibiotics and toxic compounds	Bile hydrolysis	Choloylglycine hydrolase (EC 3.5.1.24)
		Beta-lactamase	Beta-lactamase (EC 3.5.2.6)
