# Supplementary material for: Ecological Overlap and Horizontal Gene Transfer in Staphylococcus aureus and Staphylococcus epidermidis
Source: Genome Biol Evol. 2015 Apr 16;7(5):1313–28. doi: 10.1093/gbe/evv066 (PMC4453061; doi:10.1093/gbe/evv066)
Supplement: Supplementary Data [file supp_evv066_suppl_data.zip › Table S3.pdf]

Table S3. Predicted functions of genes found to be recombining in *S. aureus* but not *S. epidermidis*. The different columns labels and contents were defined by the RAST automatic annotation pipeline.

Category	Sub-category	Sub-system	Role
Amino Acids and Derivatives	Glutamine, glutamate, aspartate, asparagine; ammonia assimilation	Glutamine, Glutamate, Aspartate and Asparagine Biosynthesis	Glutamine synthetase type I (EC 6.3.1.2)
			Glutamate synthase [NADPH] small chain (EC 1.4.1.13)
			Ferredoxin-dependent glutamate synthase (EC 1.4.7.1)
			L-asparaginase (EC 3.5.1.1)
		Glutamate dehydrogenases	NAD-specific glutamate dehydrogenase (EC 1.4.1.2)
		Glutamine synthetases	Glutamine synthetase type I (EC 6.3.1.2)
	Arginine; urea cycle, polyamines	Arginine and Ornithine Degradation	Arginine decarboxylase (EC 4.1.1.19)
			Arginine deiminase (EC 3.5.3.6)
	Lysine, threonine, methionine, and cysteine	Methionine Transport	Methionine ABC transporter substrate-binding protein
			Methionine ABC transporter ATP-binding protein
			Methionine ABC transporter permease protein
		Methionine Degradation	S-adenosylmethionine synthetase (EC 2.5.1.6)
			Cystathionine gamma-lyase (EC 4.4.1.1)
		Methionine Biosynthesis	5,10-methylenetetrahydrofolate reductase (EC 1.5.1.20)
			5-methyltetrahydropteroyltriglutamate--homocysteine methyltransferase (EC 2.1.1.14)
		Threonine degradation	Low-specificity L-threonine aldolase (EC 4.1.2.5)
		Lysine degradation	Lysine decarboxylase (EC 4.1.1.18)
	Aromatic amino acids and derivatives	Common Pathway For Synthesis of Aromatic Compounds (DAHP synthase to chorismate)	5-Enolpyruvylshikimate-3-phosphate synthase (EC 2.5.1.19)
			2-keto-3-deoxy-D-arabino-heptulosonate-7-phosphate synthase I beta (EC 2.5.1.54)
		Phenylalanine and Tyrosine Branches from Chorismate	Chorismate mutase I (EC 5.4.99.5)
	Alanine, serine, and glycine	Glycine Biosynthesis	Low-specificity L-threonine aldolase (EC 4.1.2.5)
Carbohydrates	Central carbohydrate metabolism	Pyruvate Alanine Serine Interconversions	Branched-chain amino acid aminotransferase (EC 2.6.1.42)
			Alanine dehydrogenase (EC 1.4.1.1)
	Di- and oligosaccharides	Sucrose utilization	Sucrose-6-phosphate hydrolase (EC 3.2.1.26)
			Sucrose operon repressor ScrR, LacI family
			PTS system, sucrose-specific IIC component (EC 2.7.1.69)
			PTS system, sucrose-specific IIB component (EC 2.7.1.69)
			Fructokinase (EC 2.7.1.4)
	One-carbon Metabolism	One-carbon metabolism by tetrahydropterines	5,10-methylenetetrahydrofolate reductase (EC 1.5.1.20)
	Organic acids	Alpha-acetolactate operon	Alpha-acetolactate decarboxylase (EC 4.1.1.5)
			Acetolactate synthase, catabolic (EC 2.2.1.6)
	Fermentation	Butanol Biosynthesis	Alcohol dehydrogenase (EC 1.1.1.1)
			Pyruvate formate-lyase (EC 2.3.1.54)
			Butyryl-CoA dehydrogenase (EC 1.3.8.1)
			Acetyl-CoA acetyltransferase (EC 2.3.1.9)
		Fermentations: Lactate	Phosphate acetyltransferase (EC 2.3.1.8)
		Acetoin, butanediol metabolism	Alpha-acetolactate decarboxylase (EC 4.1.1.5)
			Acetolactate synthase large subunit (EC 2.2.1.6)
			Acetolactate synthase, catabolic (EC 2.2.1.6)
	Monosaccharides	Mannose Metabolism	Phosphomannomutase (EC 5.4.2.8)
			Mannose-6-phosphate isomerase, class I (EC 5.3.1.8)
Cell Wall and Capsule	Cell Wall and Capsule - no subcategory	Recycling of Peptidoglycan Amino Sugars	N-acetylmuramic acid 6-phosphate etherase (EC 4.2.-.-)
	Gram-Positive cell wall components	Sortase	Cell surface protein IsdA, transfers heme from hemoglobin to apo-IsdC
		Polyglycerolphosphate lipoteichoic acid biosynthesis	Lipoteichoic acid synthase LtaS Type Ib
Cofactors, Vitamins, Prosthetic Groups, Pigments	Lipoic acid	Lipoic acid metabolism	Lipoate-protein ligase A

			Lipoate synthase
DNA Metabolism	DNA repair	DNA repair, bacterial	Methylated-DNA--protein-cysteine methyltransferase (EC 2.1.1.63)
			Endonuclease IV (EC 3.1.21.2)
			SOS-response repressor and protease LexA (EC 3.4.21.88)
		DNA repair, UvrABC system	Excinuclease ABC subunit C
		DNA repair, UvrABC system	Excinuclease ABC subunit A
		DNA repair, bacterial photolyase	Deoxyribodipyrimidine photolyase (EC 4.1.99.3)
		DNA repair, bacterial DinG and relatives	DinG family ATP-dependent helicase YoaA
		DNA repair, bacterial UvrD and related helicases	ATP-dependent DNA helicase UvrD/PcrA
		ATP-dependent Nuclease	ATP-dependent nuclease, subunit B
			ATP-dependent nuclease, subunit A
	DNA uptake, competence	DNA processing cluster	FIG000557: hypothetical protein co-occurring with RecR
			DNA polymerase III subunits gamma and tau (EC 2.7.7.7)
Fatty Acids, Lipids, and Isoprenoids	Phospholipids	Cardiolipin synthesis	Cardiolipin synthetase (EC 2.7.8.-)
	Isoprenoids	Mevalonate Branch of Isoprenoid Biosynthesis	Diphosphomevalonate decarboxylase (EC 4.1.1.33)
			Hydroxymethylglutaryl-CoA synthase (EC 2.3.3.10)
			Hydroxymethylglutaryl-CoA reductase (EC 1.1.1.34)
			Acetyl-CoA acetyltransferase (EC 2.3.1.9)
		Isoprenoinds for Quinones	Octaprenyl diphosphate synthase (EC 2.5.1.90)
			Dimethylallyltransferase (EC 2.5.1.1)
			Geranylgeranyl diphosphate synthase (EC 2.5.1.29)
			Heptaprenyl diphosphate synthase component II (EC 2.5.1.30)
		Isoprenoid Biosynthesis: Interconversions	(2E,6E)-farnesyl diphosphate synthase (EC 2.5.1.10)
Iron acquisition and metabolism	Iron acquisition and metabolism - no subcategory	Ferrous iron transporter EfeUOB, low-pH-induced	Ferrous iron transport permease EfeU
Membrane Transport	ABC transporters	ABC transporter dipeptide (TC 3.A.1.5.2)	Dipeptide-binding ABC transporter, periplasmic substrate-binding component (TC 3.A.1.5.2)
	Protein translocation across cytoplasmic membrane	Twin-arginine translocation system	Twin-arginine translocation protein TatCd
	Cation transporters	Magnesium transport	Mg/Co/Ni transporter MgtE
Nitrogen Metabolism	Nitrogen Metabolism - no subcategory	Nitrate and nitrite ammonification	Respiratory nitrate reductase alpha chain (EC 1.7.99.4)
			Nitrate/nitrite transporter
		Ammonia assimilation	Ammonium transporter
Nucleosides and Nucleotides	Purines	GMP synthase	GMP synthase [glutamine-hydrolyzing], ATP pyrophosphatase subunit (EC 6.3.5.2)
			GMP synthase [glutamine-hydrolyzing], amidotransferase subunit (EC 6.3.5.2)
	Nucleosides and Nucleotides - no subcategory	dNTP triphosphohydrolase protein family	dNTP triphosphohydrolase, putative
Protein Metabolism	Protein biosynthesis	tRNA aminoacylation, Pro	Prolyl-tRNA synthetase (EC 6.1.1.15), bacterial type
		tRNA aminoacylation, Trp	Tryptophanyl-tRNA synthetase (EC 6.1.1.2)
	Protein degradation	Metalloendopeptidases (EC 3.4.24.-)	Glycyl-glycine endopeptidase LytM precursor (EC 3.4.24.75)
		Aminopeptidases (EC 3.4.11.-)	Cytosol aminopeptidase PepA (EC 3.4.11.1)
		Omega peptidases (EC 3.4.19.-)	Pyrrolidone-carboxylate peptidase (EC 3.4.19.3)
Regulation and Cell signaling	Regulation and Cell signaling - no subcategory	Cell envelope-associated LytR-CpsA-Psr transcriptional attenuators	Cell envelope-associated transcriptional attenuator LytR-CpsA-Psr, subfamily F2 (as in PMID19099556)
Respiration	Electron accepting reactions	Anaerobic respiratory reductases	Butyryl-CoA dehydrogenase (EC 1.3.8.1)
RNA Metabolism	RNA processing and modification	RNA pseudouridine syntheses	tRNA pseudouridine synthase A (EC 4.2.1.70)
		Ribonucleases	Ribonuclease HII (EC 3.1.26.4)
			Ribonuclease J2 (endoribonuclease in RNA processing)
			Ribonuclease HIII (EC 3.1.26.4)
		RNA processing and degradation, bacterial	3'-to-5' exoribonuclease RNase R
	Transcription	Transcription factors bacterial	Transcription accessory protein (S1 RNA-binding domain)
			Transcription-repair coupling factor
Stress Response	Oxidative stress	Flavohaemoglobin	Flavohemoprotein (Hemoglobin-like protein) (Flavohemoglobin) (Nitric oxide dioxygenase) (EC 1.14.12.17)
	Cold shock	Cold shock, CspA family of proteins	Cold shock protein CspC
Sulfur Metabolism	Sulfur Metabolism - no subcategory	Thioredoxin-disulfide reductase	Alkyl hydroperoxide reductase protein F (EC 1.6.4.-)

			Thioredoxin reductase (EC 1.8.1.9)
Virulence, Disease and Defense	Bacteriocins, ribosomally synthesized antibacterial peptides	Bacitracin Stress Response	Two-component response regulator BceR
			Bacitracin export permease protein BceB
	Resistance to antibiotics and toxic compounds	Mercury resistance operon	Mercuric ion reductase (EC 1.16.1.1)
		Multidrug Resistance Efflux Pumps	Multi antimicrobial extrusion protein (Na(+)/drug antiporter), MATE family of MDR efflux pumps
			Acriflavin resistance protein
		Mercuric reductase	PF00070 family, FAD-dependent NAD(P)-disulphide oxidoreductase
