# Supplementary material for: Ecological Overlap and Horizontal Gene Transfer in Staphylococcus aureus and Staphylococcus epidermidis
Source: Genome Biol Evol. 2015 Apr 16;7(5):1313–28. doi: 10.1093/gbe/evv066 (PMC4453061; doi:10.1093/gbe/evv066)
Supplement: Supplementary Data [file supp_evv066_suppl_data.zip › Table S4.pdf]

Table S4. Predicted functions of genes found to be recombining in *S. epidermidis* but not *S. aureus*. The different columns labels and contents were defined by the RAST automatic annotation pipeline.

Category	Sub-category	Sub-system	Role
Amino Acids and Derivatives	Arginine; urea cycle, polyamines	Arginine Deiminase Pathway	Arginine pathway regulatory protein ArgR, repressor of arg regulon
			Transcriptional regulator ArcR essential for anaerobic expression of the ADI pathway, Crp/Fnr family
			Ornithine carbamoyltransferase (EC 2.1.3.3)
		Arginine and Ornithine Degradation	Arginine/ornithine antiporter ArcD
			Arginine pathway regulatory protein ArgR, repressor of arg regulon
			Arginine decarboxylase (EC 4.1.1.19)
			Transcriptional regulator ArcR essential for anaerobic expression of the ADI pathway, Crp/Fnr family
			Arginase (EC 3.5.3.1)
			Ornithine carbamoyltransferase (EC 2.1.3.3)
	Lysine, threonine, methionine, and cysteine	Threonine degradation	Threonine dehydrogenase and related Zn-dependent dehydrogenases
		Cysteine Biosynthesis	Cysteine synthase (EC 2.5.1.47)
			Sulfite reductase [NADPH] hemoprotein beta-component (EC 1.8.1.2)
			Cystathionine gamma-lyase (EC 4.4.1.1)
			Adenylylsulfate kinase (EC 2.7.1.25)
			Serine acetyltransferase (EC 2.3.1.30)
			Cystathionine beta-synthase (EC 4.2.1.22)
		Lysine degradation	Lysine decarboxylase (EC 4.1.1.18)
	Aromatic amino acids and derivatives	Common Pathway For Synthesis of Aromatic Compounds (DAHP synthase to chorismate)	Shikimate kinase I (EC 2.7.1.71)
		Chorismate: Intermediate for synthesis of Tryptophan, PAPA antibiotics, PABA, 3-hydroxyanthranilate and more.	Phosphoribosylanthranilate isomerase (EC 5.3.1.24)
	Alanine, serine, and glycine	Serine Biosynthesis	D-3-phosphoglycerate dehydrogenase (EC 1.1.1.95)
Carbohydrates	Central carbohydrate metabolism	Methylglyoxal Metabolism	Aldehyde dehydrogenase B (EC 1.2.1.22)
			Aldehyde dehydrogenase (EC 1.2.1.3)
			Hydroxyacylglutathione hydrolase (EC 3.1.2.6)
			Aldehyde dehydrogenase A (EC 1.2.1.22)
		Pyruvate metabolism II: acetyl-CoA, acetogenesis from pyruvate	Aldehyde dehydrogenase (EC 1.2.1.3)
			Acetaldehyde dehydrogenase (EC 1.2.1.10)
			Acetate kinase (EC 2.7.2.1)
			Pyruvate decarboxylase (EC 4.1.1.1)
			Acylphosphate phosphohydrolase (EC 3.6.1.7), putative
		Glycolysis and Gluconeogenesis	6-phosphofructokinase (EC 2.7.1.11)
			Triosephosphate isomerase (EC 5.3.1.1)
			Pyruvate,phosphate dikinase (EC 2.7.9.1)
			Glucose-6-phosphate isomerase (EC 5.3.1.9)
			Fructose-bisphosphate aldolase class I (EC 4.1.2.13)
			NADPH-dependent glyceraldehyde-3-phosphate dehydrogenase (EC 1.2.1.13)
			Glucokinase (EC 2.7.1.2)
			Phosphoglycerate mutase (EC 5.4.2.1)
			Phosphoglycerate kinase (EC 2.7.2.3)
		Glycolate, glyoxylate interconversions	Glyoxylate reductase (EC 1.1.1.26)
			Hydroxypyruvate reductase (EC 1.1.1.81)
			Glyoxylate reductase (EC 1.1.1.79)
	Aminosugars	Chitin and N-acetylglucosamine utilization	Glucosamine-6-phosphate deaminase (EC 3.5.99.6)
			N-acetylglucosamine-6-phosphate deacetylase (EC 3.5.1.25)

	One-carbon Metabolism	Formaldehyde assimilation: Ribulose monophosphate pathway	6-phospho-3-hexuloisomerase
	Organic acids	Glycerate metabolism	Hydroxypyruvate reductase (EC 1.1.1.81)
			Glycerate kinase (EC 2.7.1.31)
	Fermentation	Fermentations: Lactate	D-lactate dehydrogenase (EC 1.1.1.28)
			Acetaldehyde dehydrogenase (EC 1.2.1.10)
			Acetate kinase (EC 2.7.2.1)
		Acetyl-CoA fermentation to Butyrate	3-hydroxyacyl-CoA dehydrogenase (EC 1.1.1.35)
			Acetyl-CoA acetyltransferase (EC 2.3.1.9)
	Monosaccharides	D-ribose utilization	Ribose operon repressor
			Ribokinase (EC 2.7.1.15)
		Xylose utilization	Xylulose kinase (EC 2.7.1.17)
		D-gluconate and ketogluconates metabolism	Glucose 1-dehydrogenase (EC 1.1.1.47)
			Gluconate permease, Bsu4004 homolog
			6-phosphogluconate dehydrogenase, decarboxylating (EC 1.1.1.44)
Cell Division and Cell Cycle	Cell Division and Cell Cycle - no subcategory	YgjD and YeaZ	TsaD/Kae1/Qri7 protein, required for threonylcarbamoyladenosine t(6)A37 formation in tRNA
Cell Wall and Capsule	Capsular and extracellular polysacchrides	Sialic Acid Metabolism	PTS system, N-acetylmuramic acid-specific IIB component (EC 2.7.1.69)
			N-acetylmannosamine-6-phosphate 2-epimerase (EC 5.1.3.9)
			Glucosamine-6-phosphate deaminase (EC 3.5.99.6)
			Glucosamine--fructose-6-phosphate aminotransferase [isomerizing] (EC 2.6.1.16)
			Phosphoglucosamine mutase (EC 5.4.2.10)
			PTS system, N-acetylmuramic acid-specific IIC component (EC 2.7.1.69)
			N-acetylglucosamine-6-phosphate deacetylase (EC 3.5.1.25)
			UDP-N-acetylglucosamine 2-epimerase (EC 5.1.3.14)
	Cell Wall and Capsule - no subcategory	Murein Hydrolases	D-alanyl-D-alanine carboxypeptidase (EC 3.4.16.4)
		UDP-N-acetylmuramate from Fructose-6-phosphate Biosynthesis	UDP-N-acetylenolpyruvoylglucosamine reductase (EC 1.1.1.158)
			N-acetylglucosamine-1-phosphate uridyltransferase eukaryotic (EC 2.7.7.23)
Cofactors, Vitamins, Prosthetic Groups, Pigments	Biotin	Biotin biosynthesis	Long-chain-fatty-acid--CoA ligase (EC 6.2.1.3)
			Biotin synthase (EC 2.8.1.6)
			3-ketoacyl-CoA thiolase (EC 2.3.1.16)
			Substrate-specific component BioY of biotin ECF transporter
			Biotin operon repressor
			Biotin-protein ligase (EC 6.3.4.15)
			8-amino-7-oxononanoate synthase (EC 2.3.1.47)
		Biotin biosynthesis Experimental	Biotin synthase (EC 2.8.1.6)
			Competence protein F homolog, phosphoribosyltransferase domain
			8-amino-7-oxononanoate synthase (EC 2.3.1.47)
	Pyridoxine	Pyridoxin(Vitamin B6) Degradation Pathway	Hypothetical NagD-like phosphatase
	NAD and NADP	NAD and NADP cofactor biosynthesis global	Nicotinate phosphoribosyltransferase (EC 2.4.2.11)
			NAD kinase (EC 2.7.1.23)
			C-terminal domain of CinA type S
			ADP-ribose pyrophosphatase (EC 3.6.1.13)
			Nicotinate-nucleotide adenylyltransferase (EC 2.7.7.18)
	Coenzyme A	Coenzyme A Biosynthesis	2-dehydropantoate 2-reductase (EC 1.1.1.169)
			Ketol-acid reductoisomerase (EC 1.1.1.86)
			Pantoate--beta-alanine ligase (EC 6.3.2.1)
			Dephospho-CoA kinase (EC 2.7.1.24)
			Pantothenate kinase type II, eukaryotic (EC 2.7.1.33)
DNA Metabolism	DNA repair	Uracil-DNA glycosylase	Uracil-DNA glycosylase, family 1
	DNA replication	DNA topoisomerases, Type I, ATP-independent	DNA topoisomerase III (EC 5.99.1.2)
		DNA replication strays	Probable 5'-3' exonuclease Bsu YpcP

Dormancy and Sporulation	Dormancy and Sporulation - no subcategory	Sporulation-associated proteins with broader functions	Peptidyl-tRNA hydrolase (EC 3.1.1.29)
			RNA polymerase sporulation specific sigma factor SigH
Fatty Acids, Lipids, and Isoprenoids	Fatty acids	Fatty acid metabolism cluster	3-hydroxyacyl-CoA dehydrogenase (EC 1.1.1.35)
Membrane Transport	ABC transporters	ABC transporter oligopeptide (TC 3.A.1.5.1)	Oligopeptide transport system permease protein OppC (TC 3.A.1.5.1)
			Oligopeptide ABC transporter, periplasmic oligopeptide-binding protein OppA (TC 3.A.1.5.1)
	Cation transporters	Magnesium transport	Magnesium and cobalt transport protein CorA
		Copper Transport System	Copper-translocating P-type ATPase (EC 3.6.3.4)
Metabolism of Aromatic Compounds	Peripheral pathways for catabolism of aromatic compounds	Quinate degradation	3-dehydroquinate dehydratase I (EC 4.2.1.10)
	Metabolism of central aromatic intermediates	Salicylate and gentisate catabolism	Fumarylacetoacetate hydrolase family protein
Miscellaneous	Plant-Prokaryote DOE project	Single-Rhodanese-domain proteins	Rhodanese domain protein UPF0176, Firmicutes subgroup
	Miscellaneous - no subcategory	Phosphoglycerate mutase protein family	Phosphoglycerate mutase (EC 5.4.2.1)
		Muconate lactonizing enzyme family	O-succinylbenzoate synthase (EC 4.2.1.113)
		Broadly distributed proteins not in subsystems	Putative oxidoreductase YncB
Nitrogen Metabolism	Nitrogen Metabolism - no subcategory	Nitrosative stress	Nitric oxide-dependent regulator DnrN or NorA
Nucleosides and Nucleotides	Purines	Xanthine Metabolism in Bacteria	Xanthine phosphoribosyltransferase (EC 2.4.2.22)
		Purine conversions	Inosine-uridine preferring nucleoside hydrolase (EC 3.2.2.1)
			Adenylate kinase (EC 2.7.4.3)
			Xanthine phosphoribosyltransferase (EC 2.4.2.22)
			Adenine phosphoribosyltransferase (EC 2.4.2.7)
			5'-nucleotidase family protein in cluster with NagD-like phosphatase
			Inosine-5'-monophosphate dehydrogenase (EC 1.1.1.205)
	Detoxification	Nucleoside triphosphate pyrophosphohydrolase MazG	Nucleoside triphosphate pyrophosphohydrolase MazG (EC 3.6.1.8)
		Nudix proteins (nucleoside triphosphate hydrolases)	ADP-ribose pyrophosphatase (EC 3.6.1.13)
Phages, Prophages, Transposable elements, Plasmids	Phages, Prophages	Phage replication	DNA helicase, phage-associated
Phosphorus Metabolism	Phosphorus Metabolism - no subcategory	Polyphosphate	Exopolyphosphatase (EC 3.6.1.11)
Potassium metabolism	Potassium metabolism - no subcategory	Potassium homeostasis	Large-conductance mechanosensitive channel
			Osmosensitive K+ channel histidine kinase KdpD (EC 2.7.3.-)
Protein Metabolism	Protein folding	GroEL GroES	Heat-inducible transcription repressor HrcA
			Chaperone protein DnaJ
			Heat shock protein 60 family chaperone GroEL
	Protein biosynthesis	tRNA aminoacylation, Val	Valyl-tRNA synthetase (EC 6.1.1.9)
		tRNA aminoacylation, Thr	Threonyl-tRNA synthetase (EC 6.1.1.3)
		tRNA aminoacylation, Glu and Gln	Glutamyl-tRNA(Gln) amidotransferase subunit A (EC 6.3.5.7)
			Glutamyl-tRNA synthetase (EC 6.1.1.17)
			Glutamyl-tRNA(Gln) synthetase (EC 6.1.1.24)
		tRNA aminoacylation, Asp and Asn	Aspartyl-tRNA synthetase (EC 6.1.1.12)
			Aspartyl-tRNA(Asn) amidotransferase subunit A (EC 6.3.5.6)
		Translation elongation factor G family	Translation elongation factor G
		tRNA aminoacylation, Ser	Seryl-tRNA synthetase (EC 6.1.1.11)
	Protein processing and modification	Lipoprotein Biosynthesis	Lipoprotein signal peptidase (EC 3.4.23.36)
			Prolipoprotein diacylglyceryl transferase (EC 2.4.99.-)
		Signal peptidase	Signal peptidase I (EC 3.4.21.89)
		Signal peptidase	Lipoprotein signal peptidase (EC 3.4.23.36)
		G3E family of P-loop GTPases (metallocenter biosynthesis)	Urease accessory protein UreD
			Urease accessory protein UreE
			Urease alpha subunit (EC 3.5.1.5)
	Protein degradation	Aminopeptidases (EC 3.4.11.-)	Aminopeptidase S (Leu, Val, Phe, Tyr preference) (EC 3.4.11.24)
		Metallocarboxypeptidases (EC 3.4.17.-)	D-alanyl-D-alanine carboxypeptidase (EC 3.4.16.4)

		Proteolysis in bacteria, ATP-dependent	ATP-dependent Clp protease proteolytic subunit (EC 3.4.21.92)
Regulation and Cell signaling	Regulation and Cell signaling - no subcategory	Stringent Response, (p)ppGpp metabolism	GTP pyrophosphokinase (EC 2.7.6.5), (p)ppGpp synthetase I
	Programmed Cell Death and Toxin-antitoxin Systems	Murein hydrolase regulation and cell death	LrgA-associated membrane protein LrgB
			Autolysis histidine kinase LytS
			CidA-associated membrane protein CidB
			LysR family regulatory protein CidR
			Cytidine deaminase (EC 3.5.4.5)
			Holin-like protein CidA
Regulons	Atomic Regulons	ar-431-EC Molybdopterin-guanine dinucleotide biosynthesis	Molybdopterin-guanine dinucleotide biosynthesis protein MobA
Respiration	Electron accepting reactions	Anaerobic respiratory reductases	Arsenate reductase (EC 1.20.4.1)
	Electron donating reactions	Respiratory dehydrogenases 1	NADH dehydrogenase (EC 1.6.99.3)
			Aerobic glycerol-3-phosphate dehydrogenase (EC 1.1.5.3)
		Succinate dehydrogenase	Succinate dehydrogenase cytochrome b558 subunit
			Succinate dehydrogenase flavoprotein subunit (EC 1.3.99.1)
	Respiration - no subcategory	Quinone oxidoreductase family	Putative oxidoreductase YncB
RNA Metabolism	RNA processing and modification	RNA processing and degradation, bacterial	Ribonuclease III (EC 3.1.26.3)
		RNA methylation	FIG011178: rRNA methylase
			tRNA (adenine37-N(6))-methyltransferase TrmN6 (EC 2.1.1.223)
			LSU m5C1962 methyltransferase RlmI
			SSU rRNA (adenine(1518)-N(6)/adenine(1519)-N(6))-dimethyltransferase (EC 2.1.1.182)
			Ribosomal RNA large subunit methyltransferase N (EC 2.1.1.-)
			LSU m3Psi1915 methyltransferase RlmH
			rRNA small subunit 7-methylguanosine (m7G) methyltransferase GidB
			16S rRNA (guanine(966)-N(2))-methyltransferase (EC 2.1.1.171)
			Ribosomal RNA small subunit methyltransferase C (EC 2.1.1.52)
		ATP-dependent RNA helicases, bacterial	ATP-dependent RNA helicase YqfR
		16S rRNA modification within P site of ribosome	rRNA small subunit methyltransferase H
			rRNA small subunit methyltransferase I
			Cell division protein FtsL
		Queuosine-Archaeosine Biosynthesis	Queuosine biosynthesis QueD, PTPS-I
			Inosine-uridine preferring nucleoside hydrolase (EC 3.2.2.1)
			GTP cyclohydrolase I (EC 3.5.4.16) type 2
			tRNA-guanine transglycosylase (EC 2.4.2.29)
		tRNA processing	Ribonuclease P protein component (EC 3.1.26.5)
		tRNA processing	tRNA pseudouridine synthase B (EC 4.2.1.70)
	Transcription	Transcription factors bacterial	Transcription elongation factor GreA
			Transcription antitermination protein NusG
			FIG000325: clustered with transcription termination protein NusA
			Transcription termination protein NusA
Stress Response	Osmotic stress	Choline and Betaine Uptake and Betaine Biosynthesis	Osmotically activated L-carnitine/choline ABC transporter, permease protein OpuCB
			Glycine betaine ABC transport system, ATP-binding protein OpuAA (EC 3.6.3.32)
			Osmotically activated L-carnitine/choline ABC transporter, substrate-binding protein OpuCC
			High-affinity choline uptake protein BetT
			Betaine aldehyde dehydrogenase (EC 1.2.1.8)
		Glutathione: Non-redox reactions	Hydroxyacylglutathione hydrolase (EC 3.1.2.6)
		Glutathione: Redox cycle	Glutaredoxin-like protein NrdH, required for reduction of Ribonucleotide reductase class Ib
		Glutaredoxins	Glutaredoxin-like protein NrdH, required for reduction of Ribonucleotide reductase class Ib
Virulence, Disease and Defense	Resistance to antibiotics and toxic compounds	Copper homeostasis	Copper-translocating P-type ATPase (EC 3.6.3.4)
		Cobalt-zinc-cadmium resistance	Cobalt-zinc-cadmium resistance protein
			Transcriptional regulator, MerR family
		Fosfomycin resistance	Fosfomycin resistance protein FosB

	Arsenic resistance	Arsenical resistance operon trans-acting repressor ArsD
		Arsenical pump-driving ATPase (EC 3.6.3.16)
		Arsenic efflux pump protein
		Arsenate reductase (EC 1.20.4.1)
