# Supplementary material for: Ecological Overlap and Horizontal Gene Transfer in Staphylococcus aureus and Staphylococcus epidermidis
Source: Genome Biol Evol. 2015 Apr 16;7(5):1313–28. doi: 10.1093/gbe/evv066 (PMC4453061; doi:10.1093/gbe/evv066)
Supplement: Supplementary Data [file supp_evv066_suppl_data.zip › Table S5.docx]

**Table S5**. Length distribution of core genome recombinant tracts as inferred by BratNextGen using 181 genomes of *S. aureus* and 143 genomes of *S. epidermidis*

|  | ***S. aureus*** | ***S. epidermidis*** |
| --- | --- | --- |
| Number of inferred recombinant tracts | 7,777 | 7,074 |
| Minimum size^a^ | 1 | 1 |
| 25% percentile^a^ | 222 | 593 |
| **Median size^a^** | **654** | **1,568** |
| 75% percentile^a^ | 2,229 | 3,645 |
| Maximum size^a^ | 113,020 | 233,699 |
| Mean size^a^ | 2,620 | 3,967 |
| SD^a^ | 7,307 | 10,894 |
| SEM^a^ | 82.85 | 129.5 |

1. Size in base pairs (bp)
