# Supplementary figures and images for: Ecological Overlap and Horizontal Gene Transfer in Staphylococcus aureus and Staphylococcus epidermidis
Source: Genome Biol Evol. 2015 Apr 16;7(5):1313–28. doi: 10.1093/gbe/evv066 (PMC4453061; doi:10.1093/gbe/evv066)

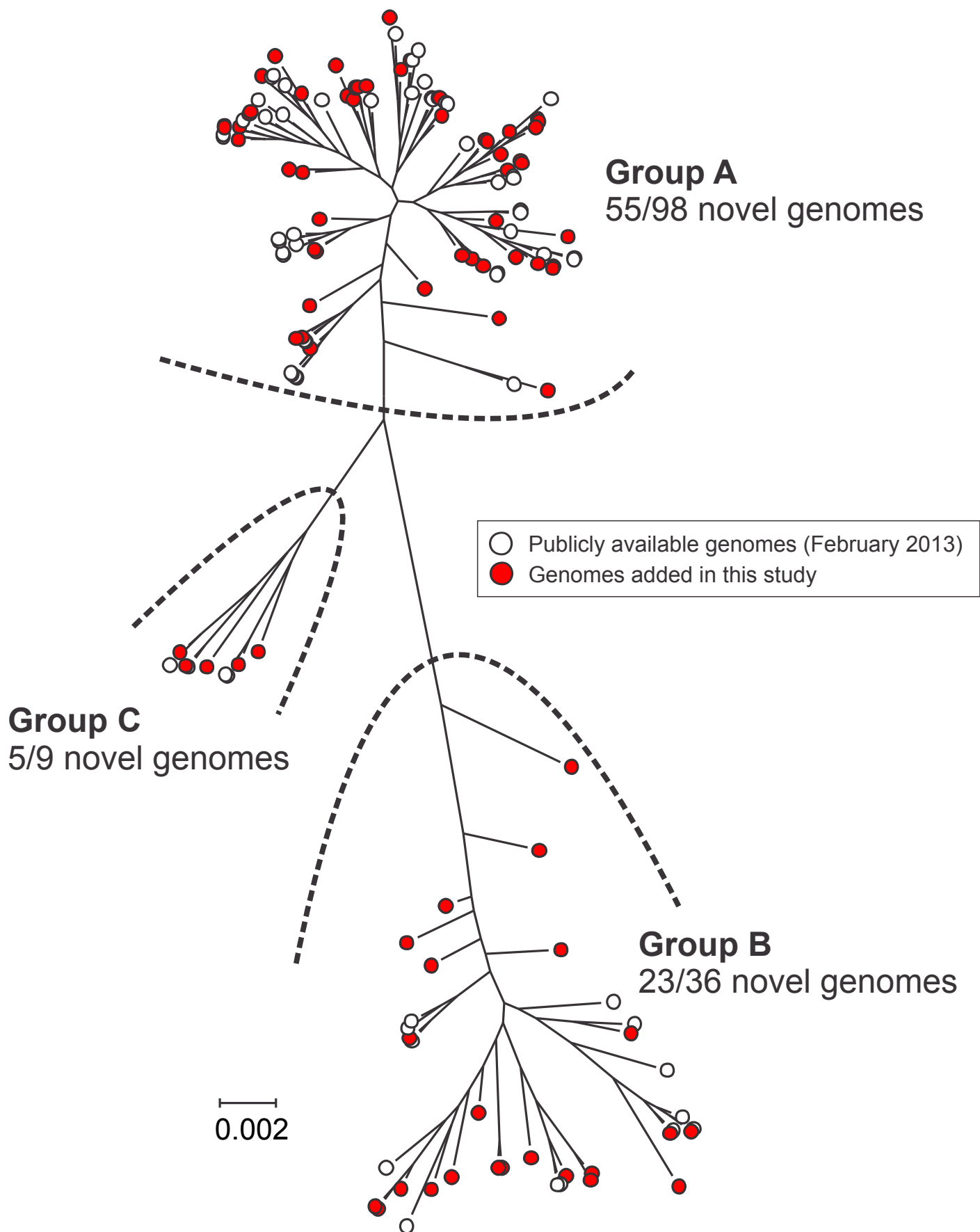

Supplement: Supplementary Data [file supp_evv066_suppl_data.zip › Figure S1.pdf]

**A**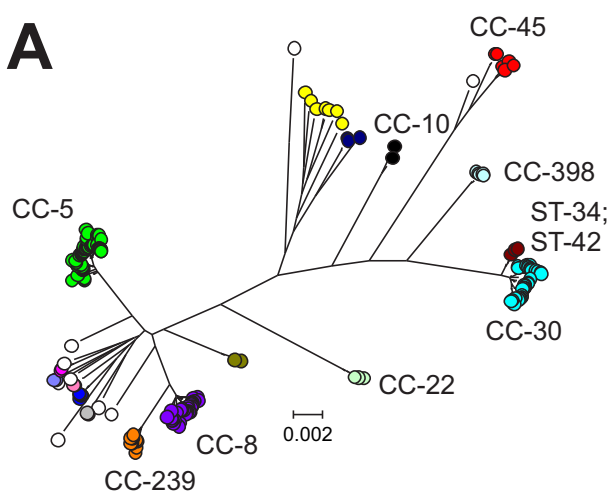**B**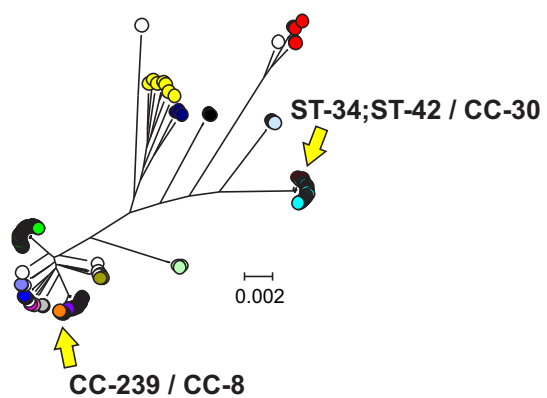**C**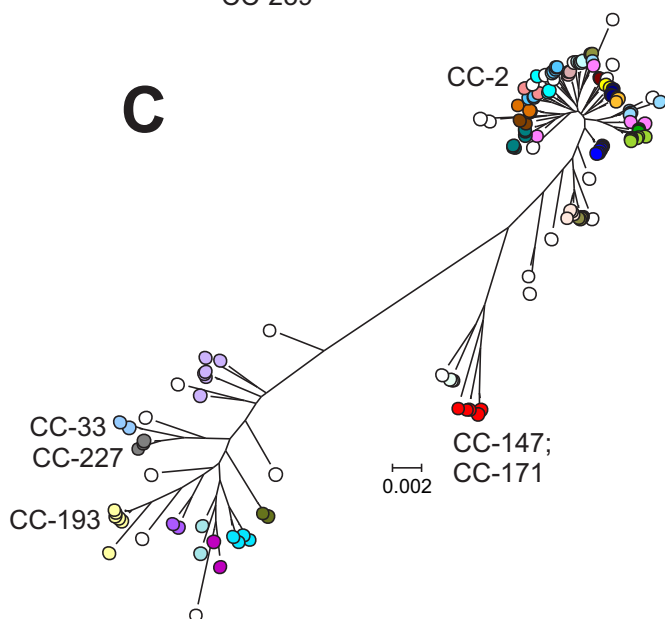**D**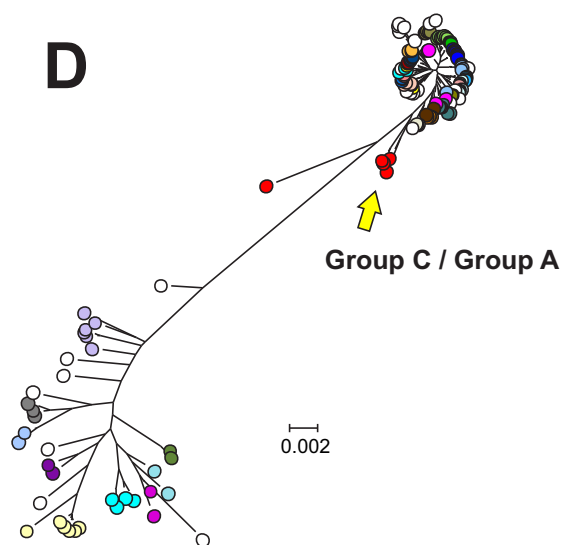

Supplement: Supplementary Data [file supp_evv066_suppl_data.zip › Figure S2.pdf]

# Metal resistance

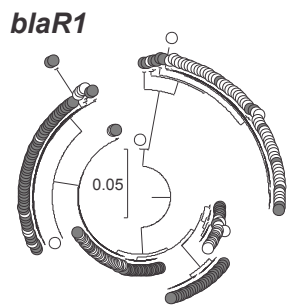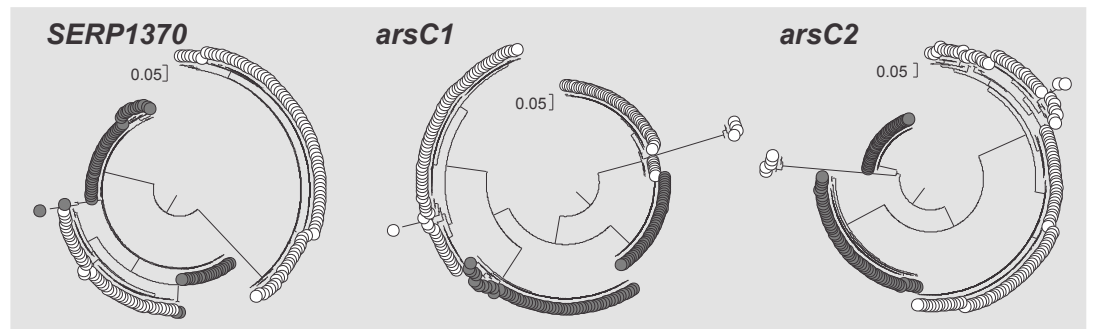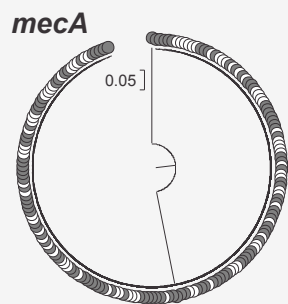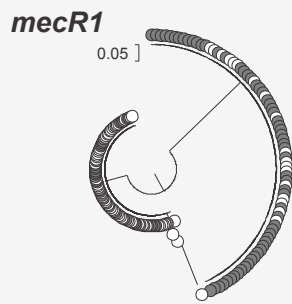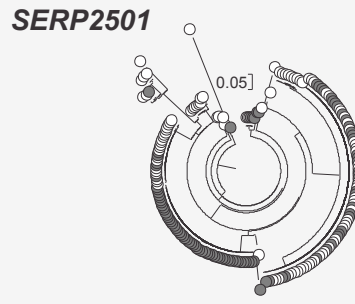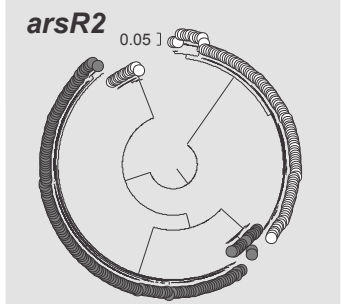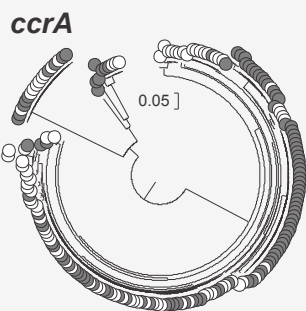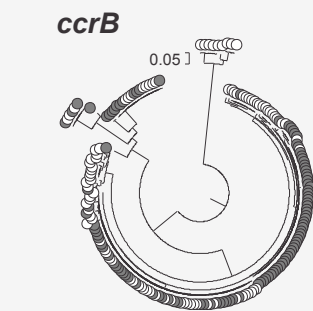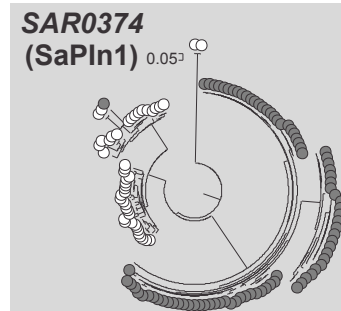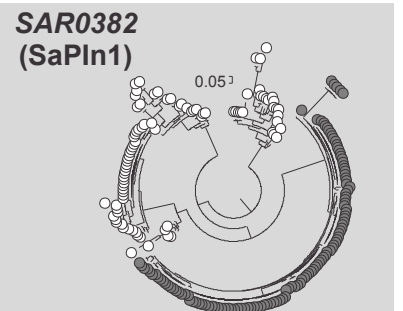

## SaPIIn1

SCCmec

Supplement: Supplementary Data [file supp_evv066_suppl_data.zip › Figure S3.pdf]

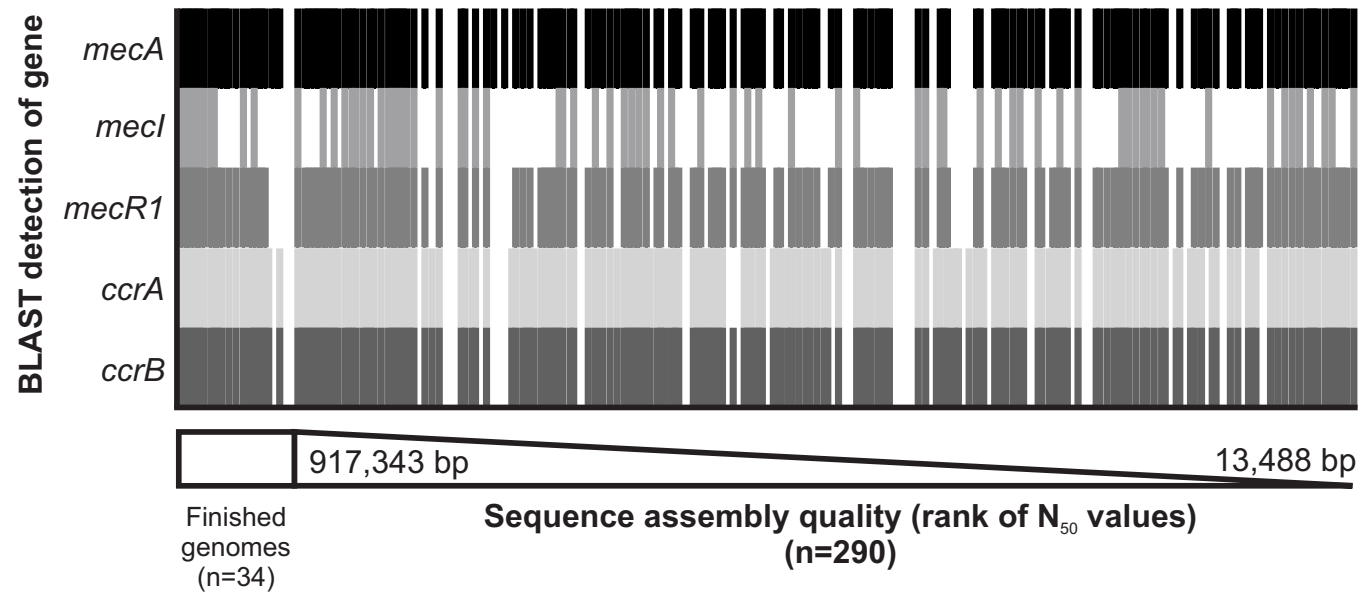

Supplement: Supplementary Data [file supp_evv066_suppl_data.zip › Figure S4.pdf]

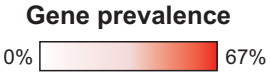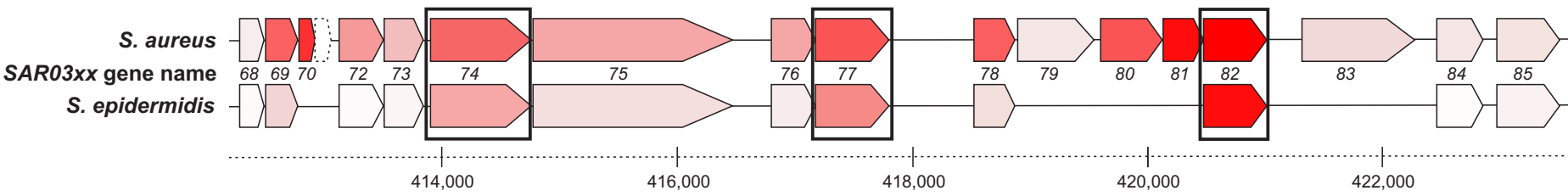

Supplement: Supplementary Data [file supp_evv066_suppl_data.zip › Figure S5.pdf]
